# Respiratory Viral Infection-Induced Microbiome Alterations and Secondary Bacterial Pneumonia

**DOI:** 10.3389/fimmu.2018.02640

**Published:** 2018-11-16

**Authors:** Shigeo Hanada, Mina Pirzadeh, Kyle Y. Carver, Jane C. Deng

**Affiliations:** ^1^Division of Pulmonary and Critical Care Medicine, University of Michigan, Ann Arbor, MI, United States; ^2^Toranomon Hospital, Tokyo, Japan; ^3^Veterans Affairs Healthcare System, Ann Arbor, MI, United States

**Keywords:** gut microbiome, respiratory viral infection, bacterial pneumonia, viral-bacterial interaction, influenza, host-microbe interaction

## Abstract

Influenza and other respiratory viral infections are the most common type of acute respiratory infection. Viral infections predispose patients to secondary bacterial infections, which often have a more severe clinical course. The mechanisms underlying post-viral bacterial infections are complex, and include multifactorial processes mediated by interactions between viruses, bacteria, and the host immune system. Studies over the past 15 years have demonstrated that unique microbial communities reside on the mucosal surfaces of the gastrointestinal tract and the respiratory tract, which have both direct and indirect effects on host defense against viral infections. In addition, antiviral immune responses induced by acute respiratory infections such as influenza are associated with changes in microbial composition and function (“dysbiosis”) in the respiratory and gastrointestinal tract, which in turn may alter subsequent immune function against secondary bacterial infection or alter the dynamics of inter-microbial interactions, thereby enhancing the proliferation of potentially pathogenic bacterial species. In this review, we summarize the literature on the interactions between host microbial communities and host defense, and how influenza, and other acute respiratory viral infections disrupt these interactions, thereby contributing to the pathogenesis of secondary bacterial infections.

## Introduction

Influenza and bacterial pneumonia are the leading cause of morbidity and mortality from infectious diseases worldwide. Influenza and other respiratory viral infections predispoxsse patients to secondary bacterial super-infections, which are frequently associated with a more severe clinical course. It is estimated that the so-called “Spanish Flu” pandemic of H1N1 influenza A virus from 1918 to 1919 resulted in more than 50 million deaths, with many caused by bacterial super-infection leading to secondary pneumonia (1–7). Even in the antibiotic era, over half of patients with severe infections in the 1957 H2N2 and 1968 H3N2 pandemics had bacterial complications (8–10). Bacterial co-infection was also detected in ~30% of cases in the 2009 H1N1 pandemic, with high mortality rates despite administration of appropriate antibiotics (11–18). Thus, it is evident that a better understanding of the pathogenesis of secondary bacterial pneumonia following viral infections is needed in order to make therapeutic strides for this devastating complication.

The mechanisms of post-viral bacterial infection are complex, comprising multifactorial processes mediated by interactions between viruses, bacteria, and the host immune system. The pathogenesis of super-infection has been attributed to direct mucosal/epithelial damage by influenza virus, increased bacterial colonization of the upper and lower respiratory tracts (URT and LRT, respectively), and dysregulation of immune responses, which all lead to increased susceptibility to secondary bacterial infections. However, emerging evidence suggests that our microbial communities residing on our mucosal surfaces likely shape the rigor of our immune responses and shape the ecological relationships between host and pathogens. Over the past 10 years, intense interest has focused on examining how the microbial communities which inhabit our bodies—which some consider to be a separate “organ system” given the sheer physical bulk, number of genes, and metabolic activities—govern the balance between health and susceptibility to diseases, including infections. This raises the possibility that disruptions in the normal microbial communities by an acute viral infection might contribute to the development of post-viral bacterial pneumonia.

The recent development of culture-independent methods of microbial identification has enabled the study of microbial communities on mucosal surfaces of the human body, referred to as “microbiota.” The microbial communities of mammalian hosts are diverse, comprised of bacterial, viruses, archaea, parasites, and fungi. The Human Microbiome Project (HMP) and other similar large-scale sequencing projects worldwide have characterized the distinct microbial communities that have adapted to the unique environmental niches within our bodies, such as the gut, skin, airways, genitourinary tract, and oral cavity. The gut microbiome, in particular, has been shown to play an integral role in shaping the immune system starting early in life, with continued influence on priming the nature and robustness of immune responses throughout one's lifetime. The respiratory tract also harbors distinct communities of microbes, with multiple discrete ecological niches (e.g., nasal cavity, oropharynx, upper airways) that vary in terms of temperature, pH, oxygen tension, mucus production, and other factors.

The effects of viral infections on both the gut and respiratory microbiome have recently undergone examination. Surprisingly, influenza infection has been found to result in significant changes in the gut microbiome, despite the lack of detectable virions in the GI tract. By comparison, the effects of viral infection on the respiratory microbiome appear to be relatively modest, but detectable. While the effects of these alterations on risk of secondary bacterial pneumonia have not been studied, potential mechanisms by which these changes might modulate susceptibility to secondary bacterial infections include alterations in the nature and magnitude of the immune response in the host (microbiome on host effects) and facilitating growth of pathogens in the absence of normal commensals (inter-microbial effects). In this article, we review the current understanding of how alterations in the microbiome following viral infection might alter host immune responses and increase susceptibility to secondary bacterial infections. Although the term “microbiome” encompasses all microbial communities, there is currently a paucity of studies on how the mycobiome (fungal microbiome) and the virome (viral microbiome) affect host defense against respiratory infections and vice-versa; thus, this review will focus on the bacterial microbiome literature.

## The gut microbiome and respiratory infections

Of the niches in the body, the gut microbial community has been the most intensively studied, with over 20,000 publications to date. While the virome and mycobiome (fungi) are also being analyzed, the bulk of the literature has focused on the bacterial component of the microbiome, and thus most of our understanding of the relation of the gut microbiome to host immunity and pathogenesis of chronic diseases comes largely from studies of the bacterial community. During health, the human gut bacterial community is diverse, with each individual harboring over 100 trillion bacteria, comprised of over 150 different species. The gastrointestinal microbiota is dominated by Firmicutes (e.g., *Lactobacillus, Bacillus*, and *Clostridium*) and Bacteroidetes (e.g., *Bacteroides*), with lower abundances of Proteobacteria (e.g., *Escherichia*) and Actinobacteria (e.g., *Bifidobacterium*) (19, 20). Wild-living mice exhibit more diverse microbiomes, with significant abundance of Proteobacteria as well as Firmicutes and Bacteroidetes (21). The gut microbiome, in addition to its metabolic functions in the host, plays an integral role in the development, instruction, and priming of the immune system. Germ-free (GF) mice (which lack microbiota) have markedly underdeveloped gut-associated lymphoid tissues, decreased number and smaller-sized Peyer's patches and mesenteric lymph nodes, and defects in antibody production, compared to specific pathogen free (SPF) mice. Not surprisingly, germ-free animals exhibit increased susceptibility to multiple types of infections, including viruses, bacteria, and parasites (22,–27). However, compared to free-living mice or laboratory animals exposed to gut flora from wild mice, SPF animals have a more limited microbial community and are also more susceptible to inflammatory diseases, with a reduced immune repertoire including deficits in memory responses (21, 28, 29).

Although an extensive discussion of the healthy gut microbiome and its impact on host immunity is beyond the scope of this review, we will highlight a few important aspects of how the intestinal bacterial community microbiome maintains a healthy host immune environment. First, bacterial metabolites generated by gut commensals contribute to the maintenance of intact epithelial integrity, regulatory T-cell development, and a relatively anti-inflammatory immune state. In particular, short-chain fatty acids (SCFAs) such as acetate, propionate, and butyrate are fermentation products of dietary fiber and carbohydrates by large intestinal bacteria (30). In addition to being a major energy source for intestinal epithelial cells, SCFAs promote the development of naive CD4^+^ T cells into regulatory T cells (31, 32), induce “tolerogenic” dendritic cells in the intestinal mucosa (33), and limit autoimmity (34, 35). At the same time, microbial metabolites are integral for promoting immune responses in the gut against pathogens, including inducing secretion of IL-18 (36) and defensins (37, 38). Thus, the products of microbiome metabolism are integral to the appropriate regulation of mucosal barrier integrity and immune homeostasis. In addition, specific members of the bacterial community have been shown to foster the proper maturation and development of the immune system. While this is still an area undergoing intense investigation, one notable example is the discovery that segmented filamentous bacteria are critical promoters of intestinal mucosal IgA production (39, 40)and Th17 cell induction (41, 42).

Dysbiosis, or an imbalance in the normal composition of the microbiome, is associated with a variety of chronic diseases, many of which are characterized by chronic inflammation or abnormal metabolism, including inflammatory bowel disease, cardiovascular disease, and diabetes. Thus, fostering appropriate levels of diversity and composition of the gut microbial community is critical for promoting health and immune homeostasis. During health, the composition of the microbiome is governed by a number of selective pressures unique to each anatomic niche, including temperature, nutrient availability, pH, oxygen tension, and the local immune environment. Short-term perturbations in the gut microenvironment caused by illness, antibiotic usage, or dietary changes (e.g., starvation) can alter the gut microbiome and subsequently lead to transient alterations in immune responses. Thus, investigating whether influenza and other respiratory viruses alter the gastrointestinal microbiome could have mechanistic implications for viral-mediated suppression of antibacterial immune responses.

### Effects of acute respiratory viral infection on gut microbiome

**Figure 1:**
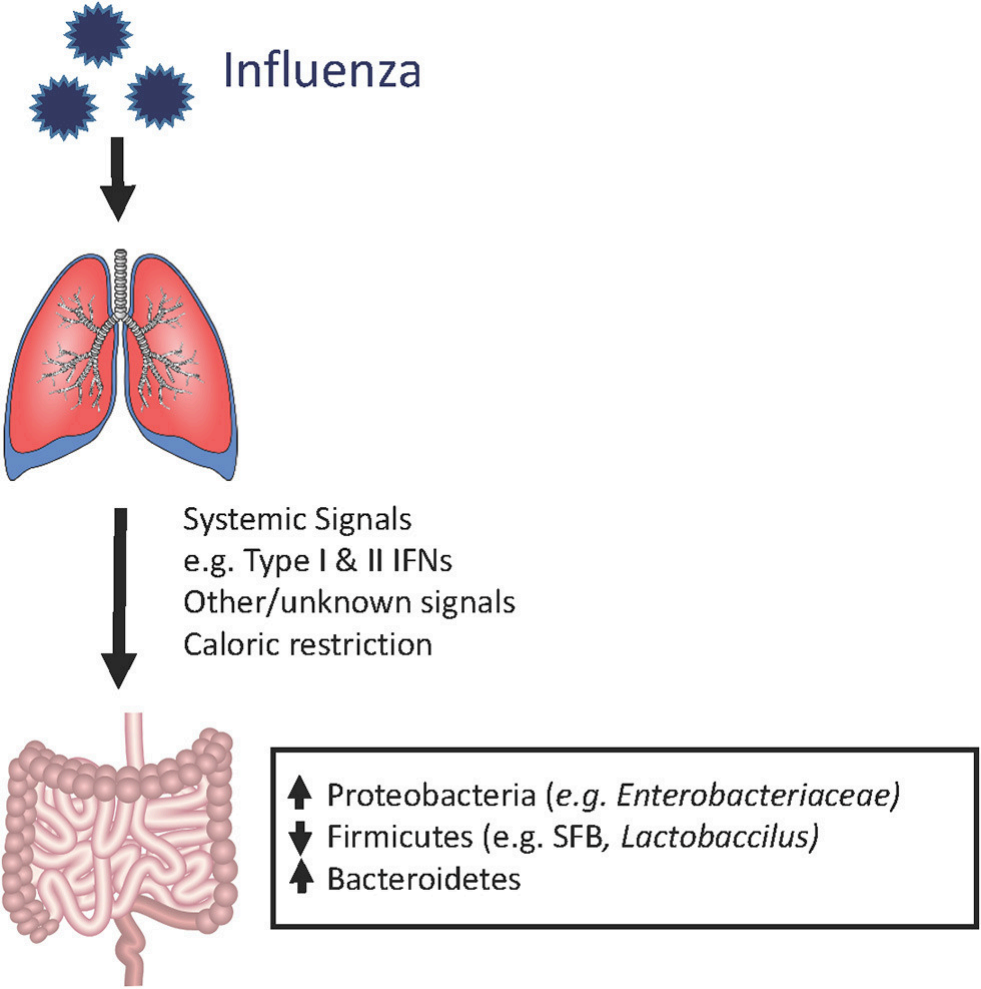
Shifts in the mouse gut microbiome in the setting of influenza infection. During an acute respiratory viral infection, changes in the bacterial composition of the gut microbiome can be observed despite the absence of detectable virus in the gastrointestinal compartment. This suggests that systemic immune signals, physiologic changes (e.g., weight loss), and other still unknown factors are disrupting the normal ecology of the gut, thereby leading to dysbiosis. However, the majority of these studies have been conducted in laboratory animals housed under SPF conditions. It remains to be determined whether human patients and mammalian hosts with more diverse baseline gut microbiota (i.e., mice in the wild), exhibit similar qualitative or quantitative changes.

Although the composition of the gastrointestinal microbiome is largely influenced by dietary patterns, respiratory viral infections could also contribute, along with other stress inducers such as broad-spectrum antibiotics exposure and chronic inflammation. Using animal models of pulmonary infections by influenza and respiratory syncytial virus (RSV), multiple groups have shown that the gut microbiome is clearly impacted by respiratory viral infections, despite the lack of detectable respiratory virus in the gut (43–47). In a murine model of influenza infection, the investigators found that although the total numbers of bacteria in the gut did not decrease, there was a reduction in the quantities of segmented filamentous bacteria (SFB) and *Lactobacillus/Lactococcus*, accompanied by increases in *Enterobacteriaceae*. Interestingly, although SFB have previously been shown to induce Th17 cells (41, 48), flu-infected mice had increased IL-17A levels and numbers of Th17 cells in the small intestine and colon, which appeared to contribute to intestinal injury (43). In this study, antibiotic treatment prior to influenza infection ameliorated the degree of intestinal injury, but not lung injury, suggesting that gut dysbiosis contributed to local but not systemic inflammation. Other groups have similarly reported increased Proteobacteria (the phylum of which *Enterobacteriaceae* are members) (44, 45), decreased Firmicutes (which include SFB, *Lactobacillus* and *Lactococcus* species), and increased Bacteroidetes (47) following infection by flu or RSVs but not after administration of live attenuated influenza vaccine (LAIV), indicating that live viral infection is required for these changes (47). The increase in Proteobacteria appears to be mediated by type I interferons (IFNs) (18), which not only depleted anaerobic bacteria but also increased susceptibility to secondary *Salmonella* colitis. However, caloric restriction also results in increased relative abundance of Proteobacteria and increased Bacteroidetes to Firmicutes ratio, raising the possibility that decreased oral intake during influenza may contribute to changes in the microbiome (45, 47, 49, 50). It has also been shown that influenza infection alters intestinal microbiota composition through type II IFN produced by lung-derived T cells recruited to the intestine (43). Thus, changes in the gut microbiome appear to result not from direct viral effects but from systemic inflammatory signals that travel from the lung and trigger local inflammatory responses in the gut (Figure 1).

### Effects of gut microbiome on host immune responses

Interactions between respiratory tract infections and the gut microbiome are bidirectional. While respiratory viral infections can change the gut microbiome, the gut microbiome also shapes the adaptive immune responses against respiratory pathogens. Mice pretreated with an antibiotic cocktail showed increased morbidity and mortality during influenza infection (51, 52). The severity of infection was associated with reductions in dendritic cell migration rate and the number of local T cells. Mice given a 4 week oral course of broad-spectrum antibiotics before respiratory viral infection mounted an attenuated anti-PR8 antibody response, were incapable of inducing CD4^+^ T cell-mediated IFN-γ response to PR8 antigen, and had fewer influenza-specific CD8^+^T cells (51, 52). These mice also had higher viral titers in their lungs (51). Germ-free mice and antibiotic-treated mice also exhibit impaired antibody responses to seasonal influenza vaccination, which was restored by oral administration of flagellated *E. coli*, demonstrating a dependence on TLR5-mediated sensing of the host microbiota (53).

**Figure 2:**
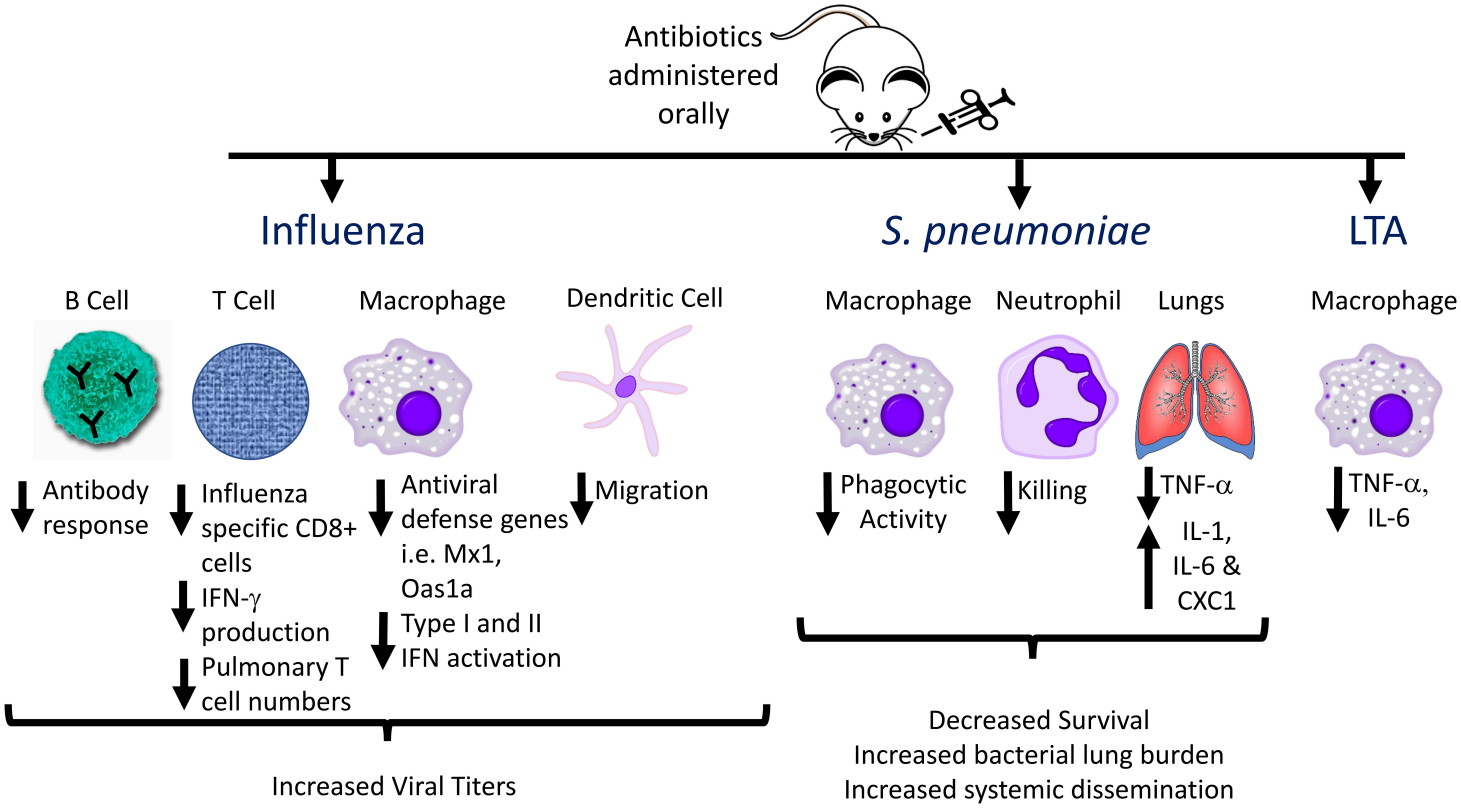
Effects of antibiotic pre-treatment on immune responses to influenza, *Streptococcus pneumoniae*, and Lipoteichoic acid (LTA). The effects of the gut microbiome on immune responses to respiratory pathogens have been investigated by administration of oral antibiotics to generate alterations in the gut flora, followed by acute infection, and analyzing host immune responses compared to non-antibiotic-pretreated animals. Multiple aspects of innate and adaptive immune responses are altered in antibiotic treated animals, including decreased antibody production, decreased phagocytic activity, and decreased inflammatory cytokine production by innate immune cells (e.g., alveolar and peritoneal macrophages) following *ex vivo* stimulation with TLR ligands.

The gut microbiome is essential for priming innate immune responses against pulmonary infections as well. During viral infections, the degree of macrophage response to respiratory viruses depends on the presence of gut microbes. Macrophages from animals treated with antibiotics exhibited defective responses to type I and II IFNs and impaired capacity to limit viral replication, suggesting that intestinal microbiota provide immune stimulation that establishes an “activation threshold” for innate antiviral immune responses (52). A comparison of C57BL/6 mice from The Jackson Laboratory (which lack SFB in the stool) and Taconic Biosciences (which are SFB positive) revealed that SFB-deficient animals have increased lung bacterial burdens and more severe pneumonia when challenged with methicillin-resistant *Staphylococcus aureus* (MRSA) (54), which was associated with decreased IL-17-mediated responses in the lung. Another study using broad-spectrum antibiotic treatment followed by intranasal administration of *S. pneumoniae* in mice demonstrated that microbiome depletion led to decreased survival, increased lung bacterial burden, and increased systemic dissemination of bacteria (55). Antibiotic-pretreated animals displayed altered cytokine profiles in the lung compared to untreated controls following *S. pneumoniae* infection, including significantly decreased TNF-α levels at 6 and 24 h after infection. Additionally, in the microbiota-depleted group, alveolar macrophages and blood neutrophils exhibited decreased phagocytic activity, and decreased inflammatory cytokine production following *ex vivo* stimulation by Toll-like receptor (TLR) ligands such as lipoteichoic acid (LTA) (55). These effects might be mediated in part by decreased Nod1 sensing of meso-DAP (diaminopimelic acid)-containing peptidoglycan found in gut microbiota, which previously was shown to be essential for priming innate immune responses to *S. pneumoniae* (56). Thus, antibiotic-induced disruptions in the normal gut microbial community alter multiple aspects of normal host defense against acute respiratory pathogens (Figure 2).

### Gut microbiome: therapeutic avenues for acute respiratory infections

Collectively, the studies above suggest that modulation of the gastrointestinal tract microbiome plays an important role in acute respiratory infections, but precisely how the microbiome should be manipulated to promote appropriate immune responses during acute respiratory infections is unclear. Currently, clinical studies have shown that although probiotics do not influence the incidence of respiratory tract infection, they do reduce the severity of symptoms and duration of the illness (57, 58). Pinpointing which members of the gut microbial community are essential for proper immune priming is challenging, but necessary for guiding further microbiome-based therapies. *Clostridium orbiscindens*, a member of the human gut microbiome, has been found to produce desaminotyrosine (DAT) from metabolism of flavonoids and amino acids. Antibiotic-treated mice exhibited markedly decreased fecal and serum DAT levels, which was associated with attenuated type I IFN responses to influenza infection and increased mortality (59). Thus, identification of DAT-producing microbiota might serve as a modality for priming type I IFN responses against viral infections. Another group demonstrated that oral administration of *Lactobacillus plantarum* enhanced the type I IFN response and lowered viral titers in the lungs in a murine model of influenza infection (60). Other *Lactobacillus* strains are known to enhance TNF-α and IFN-γ production by nasal lymphocytes upon influenza infection (61). Oral administration of a probiotic cocktail containing *Lactobacillus* restored the immune response and enhanced the activation of signaling pathways associated with recognition of single-stranded RNA virus (62). An alternative approach to administering probiotics is to alter the local metabolic environment to regulate immune responses. A recent report demonstrated that animals fed a high fiber diet had increased generation of SCFAs, leading to enhanced antiviral CD8^+^ T cell immune responses and attenuated neutrophil-mediated lung injury during influenza infection, resulting in improved survival (63). Thus, one strategy for decreasing the incidence of post-viral bacterial infections is to limit the severity of the primary viral infection.

However, activation of antiviral immune responses, including type I and type II IFNs, have been associated with increased susceptibility to secondary bacterial pneumonia (64, 65). Thus, another strategy is to enhance immune responses against common bacterial causes of pneumonia. One group re-colonized antibiotic-treated or germ-free mice with groups of cultivatable commensal bacteria, and found that administration of *Lactobacillus reuteri, Enterococcus faecalis, Lactobacillus crispatus*, and *Clostridium orbiscindens*, which are strong stimulators of NOD2 (i.e., cytosolic receptor for muramyl dipeptide, which is found in cell walls of certain bacteria), are able to protect against bacterial pneumonia by enhancing GM-CSF production (66). Whether viral-induced changes in the gut microbiome is associated with immune defects that promote secondary bacterial pneumonia, or whether the impaired antibacterial defenses observed in virally-infected hosts can be restored by augmenting certain components of the microbiome are important areas to be investigated.

## The respiratory tract microbiome

The microbiome of the respiratory tract has also been investigated in the context of viral infections. Its role in the development of secondary bacterial pneumonia following influenza and other acute respiratory viral infections is unclear. The respiratory tract is the main site of continuous contact with exogenous microbes. As is the case with the gut, immunity at the mucosal interface of the respiratory tract is a constant balance of tolerance of commensal and non-invasive microbes and immune activation against pathogens. The URT and LRT have similar microbial community compositions, although microbe densities are much higher in the former in healthy hosts. Several factors are known to influence airway microbiome composition including infection history, age, genetics, and structural lung disease.

The URT is an interconnected system consisting of the anterior nares, nasal cavity, nasopharynx, sinuses, Eustachian tube, middle ear cavity, oral cavity, oropharynx, and larynx, each of which serve as distinct niches with their own microbial communities. In healthy adults, bacteria present in the nasal cavity are typically those associated with skin, predominantly members of the Actinobacteria (e.g., *Corynebacterium* spp., *Propionibacterium* spp.), followed by Firmicutes (e.g., *Staphylococcus* spp.), and Proteobacteria (67–69). The oropharynx contains members of Firmicutes, Proteobacteria, and Bacteroidetes, including *Streptococcus, Neisseria, Haemophilus*, and *Lachnospira* spp. (68, 70, 71). Skin and oral cavity lineages are represented in the nasopharynx—e.g., *Streptococcus, Staphylococcus, Corynebacterium*, and *Prevotella* (70, 72, 73). A limited number of pathogens including *Streptococcus pneumoniae, Neisseria meningitides*, and *Haemophilus influenzae* are commensal bacteria of the URT.

In healthy individuals, the microbial community richness (i.e., the total number of bacterial taxa) is lower in the LRT than that in the URT (70, 74–76). Contrary to dogma that normal healthy lungs are a sterile environment, a distinct, and somewhat dynamic lung microbiome can be identified using sequencing technology, with microaspiration serving as the primary route of microbial immigration from the URT to the LRT (76, 77). The major phyla in healthy lungs are Bacteroidetes and Firmicutes, which mainly include *Prevotella, Veillonella*, and *Streptococcus* (78–80). Individuals with chronic airway diseases (e.g., cystic fibrosis, COPD) have increased bacterial populations in the lungs (77) and differences in the relative abundance of certain species (81). Impaired airway clearance due to intrinsic or extrinsic factors leads to the proliferation of bacterial species that can exploit this growth opportunity (82). How respiratory viral infection affects the diversity of microbial communities and whether viral-induced dysbiosis influences immune functions is being examined. Nonetheless, bacterial colonization of the URT is generally considered as the first step in the development of invasive bacterial infections (83, 84), including secondary bacterial infections following respiratory viral infection. Bacterial abundance, species diversity, and factors that shape the immune response to subsequent infections are discussed in greater detail below.

### Studies of the URT microbiome during respiratory viral infection

Respiratory viruses enter the human body through the URT and are the most common type of acute infections of the respiratory tract. One possible mechanism by which influenza and other viral infections might predispose infected hosts to secondary bacterial pneumonia is by altering the microbial composition of the upper respiratory tract, fostering enhanced growth of pathogens, and facilitating the subsequent entry of large bacterial loads into the LRT (85). This section will examine recent literature on how acute respiratory viral infections have changed the URT microbiome.

#### Cross-sectional studies

Given the effects of viruses on enhancing bacterial adherence to the epithelium (86–88), it is perhaps not surprising that multiple studies of human subjects as well as in animal models have shown that viral infections are associated with increased colonization by potentially pathogenic bacteria (known as “pathobionts”). A comparative analysis using qPCR to detect specific bacteria in adult patients with or without influenza A infection showed that *Staphylococcus aureus, S. pneumoniae*, and *H. influenzae* were present in 12, 24, and 32% of infected patients, respectively as compared to 5, 11, and 10% of uninfected patients (89). In experimental *in vitro* models, viral infections increase the colonization rates of various bacteria in the URT (90–95), including *S. pneumoniae* and *H. influenzae* (86–88). In children, influenza is associated with a 15-fold increase in nasopharyngeal titer of *S. pneumoniae* (96). Animal models have similarly confirmed that viral infection, particularly influenza, increases bacterial colonization rates in the URT, enhancing the risk of secondary bacterial infections (97–99). Higher pneumococcal colonization density has been linked to respiratory virus co-infection and invasive pneumococcal pneumonia, after adjusting for age and sex (85). Another case-control study comparing nasopharyngeal bacteria with and without pneumonia also found an association between nasopharyngeal load of *S. pneumoniae*—but not of *H. influenza* and *M. catarrhalis*—and viral co-infection and pneumonia (96). In addition, viral infections potentially may enhance transmission of bacteria. In a study of mice colonized with *S. pneumoniae* and then infected with influenza A virus 3 days after, *S. pneumoniae* transmission occurred only when all mice were infected with influenza and was blocked by an influenza-neutralizing antibody (95). However, while specific bacteria might gain a competitive advantage during viral infections, this does not universally translate to all bacterial taxa. A recent study of subjects with and without respiratory viral infections demonstrated lower overall bacterial reads from nasopharyngeal samples in virally-infected subjects compared with uninfected controls (100).

The relationship between acute viral infections and bacterial colonization appears to be bidirectional. Bacterial carriage or their ligands can increase or decrease viral infectivity rate, thereby positively or negatively influencing the subsequent host immune response to viral infection. Viral replication in the respiratory tract can be enhanced by exposure to *S. pneumoniae* (101). Patients harboring *S. pneumoniae* are more likely to experience subsequent acute respiratory illness episodes than those without colonization (102). In addition, bacteria present in the airways can modulate host responses against viral infection. The presence of a nasopharyngeal commensal protected mice against RSV-induced airway hyperresponsiveness. RSV-infected mice who underwent antibiotic-mediated depletion of *Streptococcus viridans* in the nasopharynx exhibited increases in number of inflammatory lymphocytes and airway hyperresponsiveness, and decreases in regulatory T cell number and transforming growth factor-β production (103). Others have shown that colonization of the URT with *S. aureus* drastically reduced influenza-induced acute lung injury and mortality in mice by recruiting a C-C chemokine receptor type 2^+^ cluster of differentiation (CD)11b^+^ monocyte subset to the lungs and inducing an M2 macrophage phenotype (104).

With the availability of next-generation 16S rRNA sequencing, microbiome-based studies have attempted to discern global patterns of change in the bacterial community of each anatomic niche during viral infections, such as changes in diversity. Diversity can be assessed using a variety of indices, such as total number of unique species of the microbiome (i.e., richness) or other measures that account for both richness and the evenness of relative abundance of the members of the community (e.g., Shannon index). Results from microbiome analyses have not demonstrated consistent changes in diversity when comparing virally infected subjects with healthy controls. This is not surprising given the variability of the subjects sampled, differences in type and severity of viral infections, type and timing of sample collection, and analysis methodology. In some studies, increased bacterial diversity appeared to be associated with influenza severity. A French study of children admitted to the hospital with influenza revealed increased diversity of the nasopharyngeal microflora with increased influenza severity (105). Children with severe influenza showed decreased relative abundance of *S. aureus* and increased abundance of *Prevotella, Streptobacillus, Porphyromonas, Granulicatella, Veillonella, Fusobacterium*, and *Haemophilus*. A recent Chinese study in patients with H7N9 avian influenza demonstrated significantly increased diversity in the oropharyngeal microbiome of H7N9-infected patients compared to healthy controls, particularly H7N9 patients with secondary bacterial pneumonia (106). Conversely, a French study of nasopharyngeal samples and a South Korean study of oropharyngeal samples from patients with acute respiratory viral infections both displayed decreases in diversity indices during viral infections compared to healthy controls (71, 100). Both studies included subjects ranging from infants to adults >80 years of age, limiting conclusions about age-related effects. Longitudinal studies conducted in healthy volunteers who underwent experimental self-innoculation with rhinovirus also failed to demonstrate significant changes in diversity of the URT microbiome, while administration of LAIV vaccine to healthy adults led to increases in diversity measures following viral challenge (73, 107). Thus, unlike other diseases where decreased diversity is considered deleterious to the host, the effects of viral infections on diversity *per se* are variable and not presently considered a good indicator of risk for complications, including secondary bacterial pneumonias.

Microbiome sequencing studies also enable investigators to identify changes in abundance among multiple bacterial taxa simultaneously, beyond just what can be cultured individually. This allows investigators to determine what groups of bacteria are changing in unison during viral infection and which are existing in competition with one another. This information may have implications for the development of probiotic therapies (as discussed below). A recent metagenomics-based study in France reported enrichment of *S. aureus, S. pneumoniae, H. influenzae, Moraxella catarrhalis* and *Klebsiella pneumoniae* in nasopharyngeal samples of subjects with confirmed respiratory viral infections compared to healthy controls (100). An examination of the oropharyngeal microbiome of pneumonia patients with and without 2009 influenza A H1N1 pandemic viral infection showed that Firmicutes (which include *Staphylococcus* and *Streptococcus* spp.) and Proteobacteria (mainly *Pseudomonas amygdali, P. fluorescens, Pseudomonas* sp. UK4, *Acinetobacter baumanii* and *A. junii*)—were significantly enriched in patients with influenza (108). Another study of patients with 2009 pandemic H1N1 influenza infection revealed that the predominant phyla of the upper respiratory tract (nasal and nasopharyngeal samples) in patients harboring pandemic H1N1 were Actinobacteria, Firmicutes, and Proteobacteria although normal controls were not included; however, the authors suggested that flu is associated with an expansion of Proteobacteria (109) which is generally less abundant in healthy hosts. These findings are supported by another group who found that *Moraxella* and *Enterobacter* spp. (which are classified as Proteobacteria) were the most highly represented bacteria in nasopharyngeal samples obtained from patients with pandemic H1N1 influenza (110). However, these studies demonstrated that there was considerable inter-subject variability, highlighting the need for longitudinal studies to decipher changes following viral infection.

Investigators have also sought to determine whether specific viruses are consistently linked to enrichment of certain bacterial taxa. In the nasopharyngeal compartment of Aboriginal and non-Aboriginal children in Australia, positive associations were detected between hRV and *S. pneumoniae, H. influenza*, and *Moraxella catarrhalis* carriage as well as between adenovirus and *M. catarrhalis* (111). Another study examining the presence of 20 respiratory viruses by PCR panel and prevalence of bacterial carriage in the nasopharynx of children found a strong positive association between *S. aureus* colonization and influenza virus (112). Moreover, *S. pneumoniae* colonization was positively associated with the presence of hRV and enteroviruses; *H. influenzae* was positively associated with hRV and RSV; and *M. catarrhalis* colonization was positively associated with coronaviruses and adenoviruses. A 16s rRNA sequencing-based study conducted in infants with acute RSV or hRV respiratory infections reported that infants with RSV had significantly higher abundance of *Staphylococcus* spp. compared to hRV-infected infants (113). An analysis of the URT bacterial content of 57 healthy asymptomatic individuals and 59 patients with influenza virus, parainfluenza, hRV, RSV, coronavirus, adenovirus, or metapneumovirus by culture-independent pyrosequencing revealed six distinct bacterial profiles—i.e., *Streptococcus* + *Prevotella* + *Veillonella, Streptococcus* + *Haemophilus* + *Neisseria, Streptococcus, Moraxella, Haemophilus*, and *Klebsiella*. These profiles, however, were not associated with virus type but were linked to the age of subjects (71).

Given that many human studies are cross-sectional in nature, it remains unclear whether post-viral bacterial pneumonias might be the result of viral infections enhancing bacterial colonization or acquisition, colonizing bacteria influencing host susceptibility to respiratory viral infections, or a combination of both. Another complicating factor particularly in cross-sectional studies examining the microbiome during viral infections is that the groups are not well-controlled and the sample numbers are relatively small considering the number of variables that could affect the respiratory tract microbiome–such as age, gender, oral hygiene and nose-picking habits, healthcare-based employment status, smoking status, medication use, exposure to small children, etc. The underlying type of viral infection, sampling timepoint after onset of infection, severity of infection, and concomittant antimicrobial usage are other confounding factors. This may underlie the highly variable and sometimes discrepant observations from microbiome studies in patients with viral infections.

#### Longitudinal studies

There have been few clinical studies comparing baseline pre- and post-infection microbiomes in otherwise healthy individuals with acute viral infections due to the difficulty of sampling before infection. However, the relatively few studies available provide insights into the dynamicity and stability of bacteria colonization patterns over time, and whether and how perturbations brought on by acute viral infections alter these patterns. In healthy children, the major phyla among nasopharyngeal microbiotas are Proteobacteria, Firmicutes, Bacteroidetes, Actinobacteria, and Fusobacteria, with *Moraxella, Haemophilus, Streptococcus, Flavobacteria, Dolosigranulum, Corynebacterium*, and *Neisseria* as predominant genera. Changes in nasopharyngeal microbiome diversity were observed across seasons, with a predominance of *Proteobacteria* and *Fusobacteria* in fall-winter and *Bacteroidetes* and *Firmicutes* in spring; these differences were independent of recent antibiotics and viral co-infection (114). However, another analysis of two nasopharyngeal washes collected 5.5–6.5 months apart from 40 children and adolescents with asthma showed no significant differences in nasopharyngeal microbiome diversity across seasons, although mean relative abundances of *Haemophilus, Moraxella, Staphylococcus*, and *Corynebacterium* varied significantly between summer and fall samples and between age groups. Moreover, in 87.5% of patients, operational taxonomic units (OTUs) in patients varied significantly between time points (115). An investigation of the frequency and seasonal variation in bacterial and viral load in asymptomatic healthcare professionals during the winter and summer months showed that of the 100 subjects tested during the winter, 34 were colonized with at least one bacterial species and 11 tested positive for at least one virus. The most frequently detected pathogens were methicillin-resistant *Staphylococcus aureus* (MRSA), *M. catarrhalis*, and coronavirus. In contrast, of the 100 subjects tested during the summer, 37 harbored at least one bacterium (mainly MRSA and *K. pneumoniae*) and four tested positive for one virus (116).

Several larger scale surveillance studies of mainly pediatric populations have examined the natural temporal patterns in bacterial colonization during viral infections. One clinical investigation assessed the presence and density of *S. pneumoniae, H. influenzae*, and *M. catarrhalis* in the nasopharynx of children during URT infection and in the healthy state, and reported that the proportion of children colonized with these bacteria was higher during infection than during asymptomatic surveillance visits. Mean density of all bacterial species was significantly higher at each visit when a virus was detected. Interestingly, the percentage of colonized children and bacterial density were also higher at asymptomatic visits in which virus was detected than at those in which virus was not detected (117). Another study of 31 families with small children using longitudinal nasal swab sampling demonstrated that rhinovirus infection was associated with increased acquisition of *S. pneumoniae* from the community as well as increased transmission of *S. pneumoniae* within the family (118).

**Figure 3:**
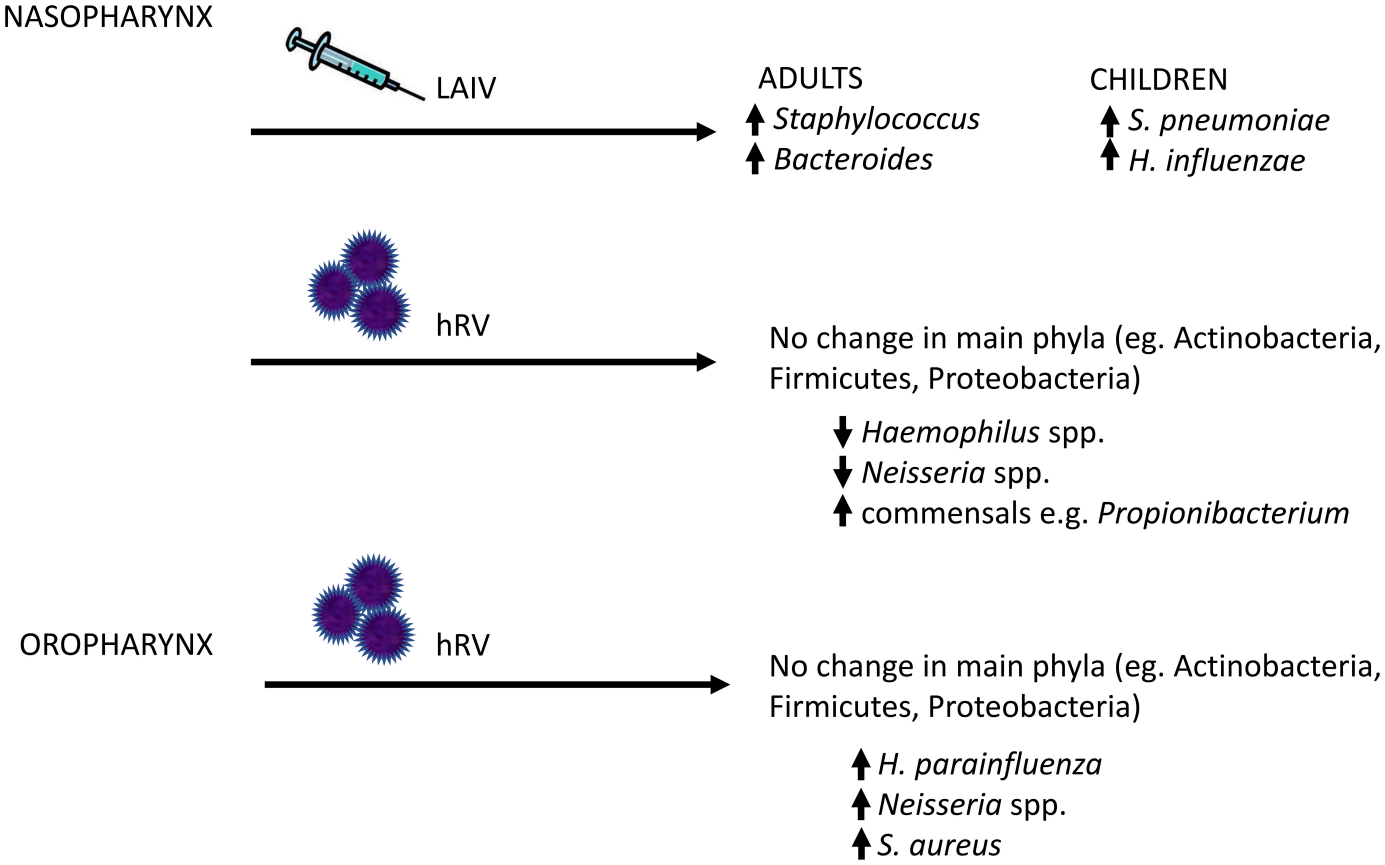
Changes in the human upper respiratory tract microbiome following viral exposure. Given that bacterial pneumonia frequently arises as a result of aspirated bacterial pathogens, a potential mechanism by which viral infections might increase the risk of secondary bacterial infections is through increased colonization of the upper respiratory tract by bacterial pathogens. In human subjects, live attenuated influenza vaccine (LAIV) and human rhinovirus (hRV) have been shown to disrupt the local host bacterial community, with increased relative abundance of potential pathogens (or pathobionts), such as Staphylococcal and Neisseria species. The major changes in the upper respiratory tract microbiome are highlighted here.

Other groups have examined the effects of experimental innoculation of hRV into the URT (nares) (Figure 3). These studies reported no significant changes in total read counts or of the main phyla (e.g., Actinobacteria, Firmicutes, and Proteobacteria) over time in nasopharyngeal samples (73) or throat swabs (119). In the oropharyngeal compartment, rhinovirus infection was associated with a strong trend toward transient increases in the relative abundances of *H. parainfluenzae, Neisseria subflava* and a weak trend toward an increase in *S. aureus* (119). By 60 days, abundance of these bacteria had returned to baseline. Nasopharyngeal sampling showed completely opposite results, with decreased relative abundance of *Haemophilus* and *Neisseria* spp., but an increase in the normal nasal commensal, *Propionibacterium*, in subjects following hRV infection (73). No differences in *Staphylococcus* were observed. However, the number of subjects were small in both studies, limiting the power to detect changes over time.

Nasopharyngeal microbiota composition has been shown to be altered by influenza vaccination (Figure 3). Administration of live attenuated influenza vaccine (LAIV), which is nasally instilled, to healthy children increased the nasal colonization density of *S. pneumoniae* in subjects who harbored this bacterium at the time of vaccination, and transiently increased rates of colonization by *H. influenza* (120). In healthy adult volunteers, it was demonstrated that intranasal LAIV administration induced an increase in the diversity of the nasopharyngeal microbiome, as well as relative abundances of *Staphylococcus* and *Bacteroides* (107). These changes were not observed in subjects given saline nasal spray. In a mouse model, bacterial density in the nasopharynx after LAIV administration was increased as much as 100,000 times compared to influenza-naive hosts, and the duration of carriage of *S. pneumoniae* or *S. aureus* was also increased 2 to 5-fold (121). However, systemic vaccination can also alter the URT microbiome. A longitudinal study of healthy subjects found a significant association between the presence of *Lactobacillus helveticus, Prevotella melaninogenica, Streptococcus infantis, Veillonella dispar*, and *Bacteroides ovatus* and influenza-specific H1 and H3 IgA antibody response (122). Thus, it is remarkable that a relatively mild viral stimulus such as flu vaccine can lead to detectable changes in the URT microbiome.

Although the data are still preliminary, animal studies have suggested that antiviral immune activation contributes to changes in the URT microbiome and facilitate colonization by potential pathogens, such as *S. aureus*. In a mouse model of *S. aureus* nasal colonization, the absence of type I IFN receptor was associated with decreased persistence of bacteria (107). Type III IFN, which is also induced during influenza infections, led to changes in the nasal microbiome, including increased numbers of culturable bacteria. Increased upper respiratory tract persistence of *S. aureus* as well as increased risk of *S. aureus* pneumonia was observed in flu-infected wildtype mice compared to mice lacking the type III IFN receptor (123). Currently, however, is it unclear to what extent viral-induced changes in the URT microbiome alter subsequent immune responses against secondary bacterial infections.

### Studies of the LRT microbiome during respiratory viral infection

Compared to studies of the URT microbiome, studies of the LRT microbiome following viral infections are relatively scarce due to the difficulty of obtaining uncontaminated samples from the lung. Samples of convenience, such as sputum, suffer from oral contamination, but bronchoscopic samples are invasive and expensive to obtain on a regular basis. Moreover, it is unclear whether outside of patients with chronic lung disease (e.g., COPD), the lung microbial burden is of sufficient magnitude to exert robust effects on immune responses and risk of secondary bacterial infection during viral infection. Data from a mouse model of influenza infection seem to indicate that flu infection has only a modest effect on bacterial counts, diversity and composition of the lung microbiome (46). In subjects with chronic obstructive pulmonary disease (COPD) after hRV infection but not in healthy individuals, there was an increase in bacterial burden and growth of bacteria present at baseline, particularly *H. influenzae* (124). The researchers observed that the growth of bacteria seemed to arise from the existing community. *S. pneumoniae* intranasally inoculated into mice pre-infected with influenza virus first colonized the nose, followed by the trachea and lungs several days later with purulent inflammation. However, this effect was not observed in uninfected animals. This suggests that pneumococcal infection may sequentially develop from the URT to the LRT in influenza virus-infected subjects (97). Thus, it is possible that some individuals with influenza infection might develop changes in their lung microbiome as a result of changes in their URT microbial communities.

Respiratory viruses not only alter the bacterial community in the URT, but also promote bacterial colonization of the LRT by a variety of mechanisms that impair bacterial clearance. First, mucus production in the respiratory tract is increased to facilitate viral clearance during infections. However, excessive mucus production can lead to airway obstruction by impeding mucociliary clearance (125). Second, viral infections can also reduce ciliary beat frequency and the number of ciliated cells, disrupt the coordinated movement of cilia, and impede the repair of respiratory epithelial cells, further leading to reduced mucociliary clearance (126, 127). Third, respiratory viral infections impair innate immune responses against bacteria (128–130). Innate immune cells including macrophages and neutrophils are recruited to the lung by cytokines and chemokines for phagocytosis and bactericidal activity. Prior viral infections dysregulate both alveolar macrophages (64, 131–136) and neutrophils (65, 128, 129), thereby inhibiting bactericidal activity. Thus, with multiple aspects of pulmonary host defense impaired, it would not be entirely surprising if a subset of influenza infected patients developed secondary bacterial pneumonia as a result of being unable to clear aspirated pathobionts from the URT.

## Intermicrobial interactions and postviral secondary infections

In addition to enabling us to determine what is present during states of health, large-scale sequencing-based microbiome analyses have also revealed who is not present during disease. It has long been appreciated that mechanisms have evolved in bacteria that confer competitive advantages, permitting them to survive in an otherwise inhospitable host environment. However, interspecies competition also maintains homeostasis of the microbial community, either through their abilities to capture scare resources (e.g., iron), or targeted killing of other bacteria (e.g., bacteriocins), preventing one microbe from dominating the community. Thus, it is possible that the immune response incited by acute viral infections, changes in the host epithelial surface caused by the virus, or the virus itself might lead to elimination of a host commensal that is responsible for keeping pathobionts in check. For example, *S. epidermidis* and *Propionibacterium acnes* abundance in the nares has been shown to be negatively associated with *S. aureus* carriage (67). Understanding these interactions may create new avenues for therapeutic interventions aimed at reducing colonization by pathogenic bacteria during influenza epidemics or pandemics.

One group of commensals that has been examined for its role in inhibiting nasal carriage by *S. aureus* and *S. pneumoniae* is *Corynebacterium* spp. An early study in Japan reported on the effects of introducing a *Corynebacterium* strain into the nares of healthy adult hospital workers who were persistent carriers of *S. aureus*, with successful eradication in 71% of subjects (137). The mechanism appeared to be bacteriocin-independent. In comparison, *S. epidermidis* implantation did not have an effect. Whether the *S. epidermidis* strain used expressed the serine protease Esp, which inhibits biofilm formation by *S. aureus* and nasal colonization (138), is unknown. Subsequent studies by another group reported that *C. pseudodiphtheriticum* inhibited *S. aureus* growth, whereas *C. accolens* and *S. aureus* appeared to support each other's growth (139). Conversely, other investigators observed that *Corynebacterium* spp. were enriched in children who were not nasally colonized with pneumococcus, and demonstrated that *C. accolens* inhibit *S. pneumoniae* growth *in vitro* by expressing a lipase that releases free fatty acids from skin surface triacylglycerols, which inhibit pneumococcal growth. Thus, painstaking identification and mechanistic interrogation of interspecies competition between commensals might lead to novel insights as to how viral infections might confer competitive advantage to pathobionts, and how to exploit natural strategies employed by commensals to restore homeostasis to the host microbial niche. Interestingly, a recent preclinical study using a murine model of RSV and *S. pneumoniae* superinfection employed nasal priming by a *C. pseudodiphtheriticum* strain to augment host defense against the viral infection, which enhanced clearance of secondary bacterial challenge and reduced lung injury measures (140).

Finally, direct effects of the infecting virus on bacteria that comprise the microbiome may facilitate the transition from pathobiont to pathogen. A metagenomic analysis showed that pH1N1-associated airway microbiotas were enriched in genes associated with cell motility, transcriptional regulation, metabolism, and response to chemotaxis compared to the same bacteria in non-infected patients (108). These data imply that influenza infection perturbs the respiratory microbiome, leading to the production of secondary metabolites including immune-modulating molecules. Viruses have also been found to impair bacterial biofilm formation and disrupt existing biofilm (141–144). Influenza has been shown to affect the *S. pneumoniae* transcriptome in terms of downregulating expression of genes associated with the colonizer state and upregulations of bacteriocins (142). Thus, direct effects of viruses on bacterial transcriptional patterns might be a mechanism by which colonizing bacteria acquire invasive potential, thereby leading to bacterial superinfections.

## Future directions

There are several areas that must be addressed by future respiratory microbiome research. First, it is necessary to standardize protocols used to analyze the respiratory microbiome, including sampling, processing, and bioinformatics methodologies. For example, sputum may be an appropriate material for investigations of respiratory diseases since it contains components of the LRT and can be obtained easily. However, more reliable information on the LRT requires invasive samples such as BAL or protected specimen brush or bronchial/lung biopsies. Second, most studies are limited to experiments conducted in animal models. Even in human studies, most analyses have been performed in a small number of patients and have described bacterial communities in the URT. The role of microbial communities outside of the lungs including gut, sinus, and skin should be considered in the context of airway diseases. Third, most studies on the microbiome have focused on the bacterial component, and have largely omitted fungi and viruses. The role of viruses—including the vast number of phages that infect bacteria—and fungi in respiratory diseases cannot be examined through 16S rRNA gene analyses, and there are no studies describing the composition and role of the respiratory virome due to the difficulty of comprehensive analyses for viruses. Fourth, it is not sufficient to study microbial communities based on species composition; a functional characterization through transcriptome and proteasome analyses is necessary to understand mechanistic role of microbiome on outcomes of infection. Finally, mucosal microbiome manipulations by vaccines, antibiotics, and probiotics in the gastrointestinal and respiratory tract niches represent novel approaches for the prevention, treatment, and management of acute and chronic lung diseases. However, given that antibiotic therapy could affect commensal bacteria and hasten the emergence of drug-resistant bacteria, more research is needed on the long-term effects of this therapy. Animal models should be developed to study the influence of the URT and LRT microbiomes on immune responses to respiratory viral infections; only then will it be possible to consider the clinical application of microbiome modulation strategies.

## Conclusion

**Figure 4:**
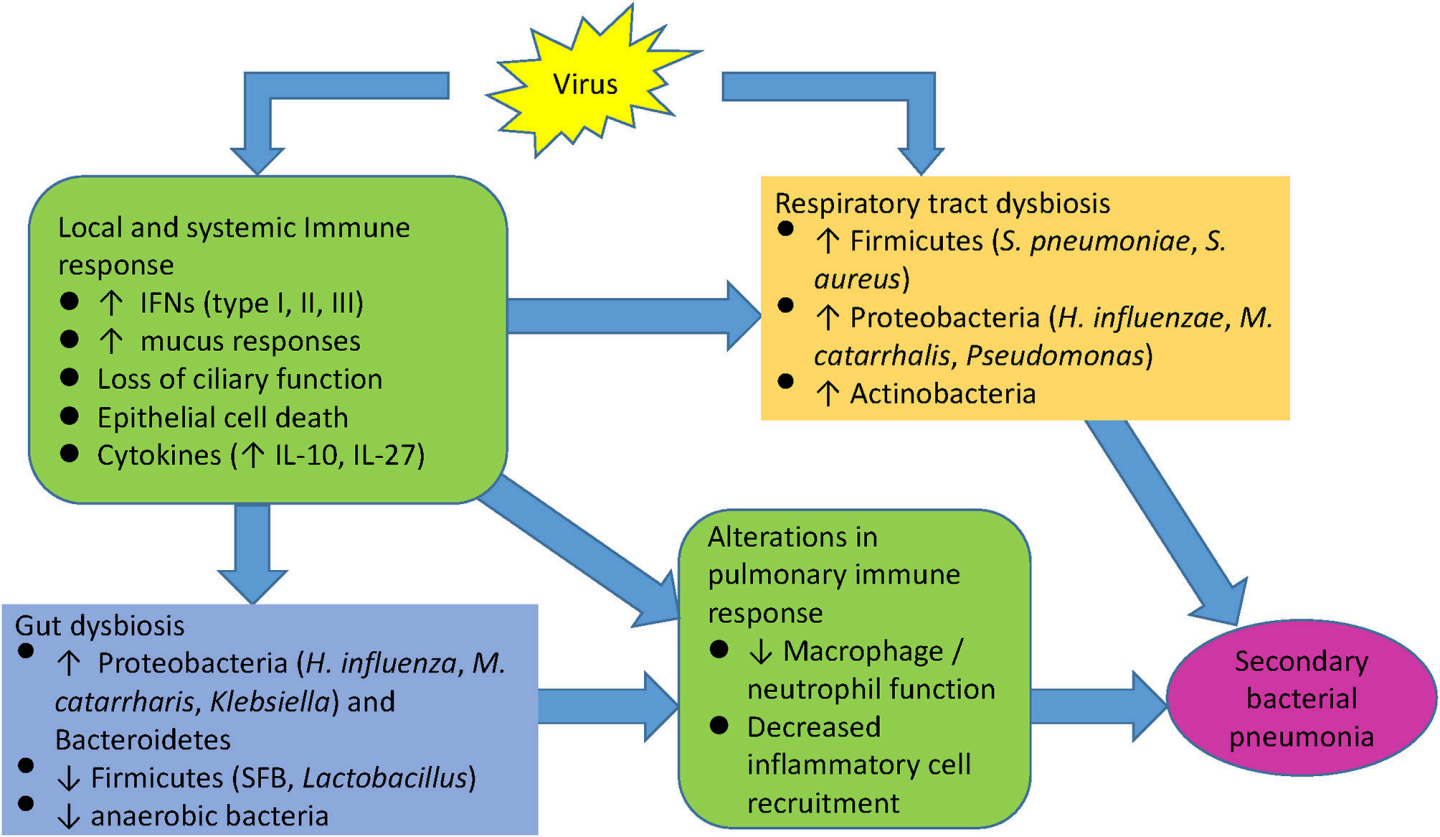
Model of viral induced susceptibility to secondary infections.

Respiratory viral infections can initiate a cascade of host immune responses that alter microbial growth conditions in the URT, LRT, and the gut (Supplemental Table 1). Activation of influenza-induced antiviral interferon pathways can lead to inadequate innate immune cell responses during host defense against secondary bacterial infections, resulting in the proliferation of potentially pathogenic bacterial species. Concomitant changes in the gut microbiome caused by the initial viral infection may also alter immune cell priming against secondary bacterial challenge, although this has not been examined to date. Although the picture is incomplete, recent microbiome literature provides additional insights into the pathogenesis of dysregulated immune responses following acute viral infections, that may promote the development of secondary bacterial pneumonias (Figure 4). Clarifying the differences and dynamics of respiratory microbiota in healthy subjects and chronic lung diseases during acute respiratory viral infections can elucidate pathogenesis of viral-bacterial interactions and provide a basis for developing novel approaches for the prevention, treatment, or management of acute respiratory infection and exacerbation of chronic lung diseases.

## Author contributions

SH and JD co-wrote the manuscript. SH, JD, and MP designed the figures and table. KC and MP edited and provided critical revisions of the manuscript. All authors approve the final version and agree to be accountable for the content of the manuscript.

### Conflict of interest statement

The authors declare that the research was conducted in the absence of any commercial or financial relationships that could be construed as a potential conflict of interest.

## References

[1] Brundage JF. Interactions between influenza and bacterial respiratory pathogens: implications for pandemic preparedness. Lancet Infect Dis. (2006) 6:303–12. 10.1016/S1473-3099(06)70466-2 16631551 PMC7106411

[2] Chien Y-W Klugman KP Morens DM. Bacterial pathogens and death during the 1918 influenza pandemic. N Engl J Med. (2009) 361:2582–3. 10.1056/NEJMc0908216 20032332

[3] Johnson NPAS Mueller J. Updating the accounts: global mortality of the 1918-1920 “Spanish” influenza pandemic. Bull Hist Med. (2002) 76:105–15. 10.1353/bhm.2002.0022 11875246

[4] McCullers JA. Insights into the interaction between influenza virus and pneumococcus. Clin Microbiol Rev. (2006) 19:571–82. 10.1128/CMR.00058-05 16847087 PMC1539103

[5] Morens DM Fauci AS. The 1918 influenza pandemic: insights for the 21st century. J Infect Dis. (2007) 195:1018–28. 10.1086/511989 17330793

[6] Morens DM Taubenberger JK Fauci AS. Predominant role of bacterial pneumonia as a cause of death in pandemic influenza: implications for pandemic influenza preparedness. J Infect Dis. (2008) 198:962–70. 10.1086/591708 18710327 PMC2599911

[7] Taubenberger JK. The origin and virulence of the 1918 “Spanish” influenza virus. Proc Am Philos Soc. (2006) 150:86–112. Available online at: http://www.jstor.org/stable/4598974 17526158 PMC2720273

[8] Bisno AL Griffin JP Van Epps KA Niell HB Rytel MW. Pneumonia and Hong Kong influenza: a prospective study of the 1968-1969 epidemic. Am J Med Sci. (1971) 261:251–63. 5092152 10.1097/00000441-197105000-00004

[9] Hers JF Masurel N Mulder J. Bacteriology and histopathology of the respiratory tract and lungs in fatal Asian influenza. Lancet (1958) 2:1141–3. 13612141 10.1016/s0140-6736(58)92404-8

[10] Oswald NC Shooter RA Curwen MP. Pneumonia complicating Asian influenza. Br Med J. (1958) 2:1305–11. 13596593 10.1136/bmj.2.5108.1305 PMC2027396

[11] Cillóniz C Ewig S Menéndez R Ferrer M Polverino E Reyes S. Bacterial co-infection with H1N1 infection in patients admitted with community acquired pneumonia. J Infect. (2012) 65:223–30. 10.1016/j.jinf.2012.04.009 22543245 PMC7132402

[12] Gill JR Sheng Z-M Ely SF Guinee DG Beasley MB Suh J. Pulmonary pathologic findings of fatal 2009 pandemic influenza A/H1N1 viral infections. Arch Pathol Lab Med. (2010) 134:235–43. 10.1043/1543-2165-134.2.235 20121613 PMC2819217

[13] Jain S Kamimoto L Bramley AM Schmitz AM Benoit SR Louie J. Hospitalized patients with 2009 H1N1 influenza in the United States, April-June 2009. N Engl J Med. (2009) 361:1935–44. 10.1056/NEJMoa0906695 19815859

[14] Kumar A Zarychanski R Pinto R Cook DJ Marshall J Lacroix J. Critically ill patients with 2009 influenza A(H1N1) infection in Canada. JAMA (2009) 302:1872–9. 10.1001/jama.2009.1496 19822627

[15] Martín-Loeches I Sanchez-Corral A Diaz E Granada RM Zaragoza R Villavicencio C. Community-acquired respiratory coinfection in critically ill patients with pandemic 2009 influenza A(H1N1) virus. Chest (2011) 139:555–62. 10.1378/chest.10-1396 20930007

[16] Palacios G Hornig M Cisterna D Savji N Bussetti AV Kapoor V. Streptococcus pneumoniae coinfection is correlated with the severity of H1N1 pandemic influenza. PLoS ONE (2009) 4:e8540. 10.1371/journal.pone.0008540 20046873 PMC2795195

[17] Shieh W-J Blau DM Denison AM Deleon-Carnes M Adem P Bhatnagar J. 2009 pandemic influenza A (H1N1): pathology and pathogenesis of 100 fatal cases in the United States. Am J Pathol. (2010) 177:166–75. 10.2353/ajpath.2010.100115 20508031 PMC2893660

[18] Domínguez-Cherit G Lapinsky SE Macias AE Pinto R Espinosa-Perez L de la Torre A. Critically Ill patients with 2009 influenza A(H1N1) in Mexico. JAMA (2009) 302:1880–7. 10.1001/jama.2009.1536 19822626

[19] Honda K Littman DR. The microbiome in infectious disease and inflammation. Annu Rev Immunol. (2012) 30:759–95. 10.1146/annurev-immunol-020711-074937 22224764 PMC4426968

[20] Human Microbiome Project Consortium Structure, function and diversity of the healthy human microbiome. Nature (2012) 486:207–14. 10.1038/nature11234 22699609 PMC3564958

[21] Rosshart SP Vassallo BG Angeletti D Hutchinson DS Morgan AP Takeda K. Wild mouse gut microbiota promotes host fitness and improves disease resistance. Cell (2017) 171:1015–28.e13. 10.1016/j.cell.2017.09.016 29056339 PMC6887100

[22] Beaman BL Gershwin ME Scates SS Ohsugi Y. Immunobiology of germfree mice infected with Nocardia asteroides. Infect Immun (1980) 29:733–43. 7011984 10.1128/iai.29.2.733-743.1980 PMC551187

[23] Inagaki H Suzuki T Nomoto K Yoshikai Y. Increased susceptibility to primary infection with Listeria monocytogenes in germfree mice may be due to lack of accumulation of L-selectin+ CD44+ T cells in sites of inflammation. Infect Immun. (1996) 64:3280–7. 8757865 10.1128/iai.64.8.3280-3287.1996 PMC174219

[24] Oliveira MR Tafuri WL Afonso LCC Oliveira MAP Nicoli JR Vieira EC. Germ-free mice produce high levels of interferon-gamma in response to infection with Leishmania major but fail to heal lesions. Parasitology (2005) 131:477–88. 10.1017/S0031182005008073 16174412

[25] Osborne LC Monticelli LA Nice TJ Sutherland TE Siracusa MC Hepworth MR. Coinfection. Virus-helminth coinfection reveals a microbiota-independent mechanism of immunomodulation. Science (2014) 345:578–82. 10.1126/science.1256942 25082704 PMC4548887

[26] Splichal I Rychlik I Gregorova D Sebkova A Trebichavsky I Splichalova A. Susceptibility of germ-free pigs to challenge with protease mutants of Salmonella enterica serovar Typhimurium. Immunobiology (2007) 212:577–82. 10.1016/j.imbio.2007.05.001 17678715

[27] Tanaka K Sawamura S Satoh T Kobayashi K Noda S. Role of the indigenous microbiota in maintaining the virus-specific CD8 memory T cells in the lung of mice infected with murine cytomegalovirus. J Immunol. (2007) 178:5209–16. 10.4049/jimmunol.178.8.5209 17404304

[28] Reese TA Bi K Kambal A Filali-Mouhim A Beura LK Bürger MC. Sequential infection with common pathogens promotes human-like immune gene expression and altered vaccine response. Cell Host Microbe (2016) 19:713–9. 10.1016/j.chom.2016.04.003 27107939 PMC4896745

[29] Beura LK Hamilton SE Bi K Schenkel JM Odumade OA Casey KA. Normalizing the environment recapitulates adult human immune traits in laboratory mice. Nature (2016) 532:512–6. 10.1038/nature17655 27096360 PMC4871315

[30] Cummings JH. Fermentation in the human large intestine: evidence and implications for health. Lancet (1983) 1:1206–9. 6134000 10.1016/s0140-6736(83)92478-9

[31] Smith PM Howitt MR Panikov N Michaud M Gallini CA Bohlooly-Y M. The microbial metabolites, short-chain fatty acids, regulate colonic Treg cell homeostasis. Science (2013) 341:569–73. 10.1126/science.1241165 23828891 PMC3807819

[32] Arpaia N Campbell C Fan X Dikiy S van der Veeken J deRoos P. Metabolites produced by commensal bacteria promote peripheral regulatory T-cell generation. Nature (2013) 504:451–5. 10.1038/nature12726 24226773 PMC3869884

[33] Goverse G Molenaar R Macia L Tan J Erkelens MN Konijn T Diet-derived short chain fatty acids stimulate intestinal epithelial cells to induce mucosal tolerogenic dendritic cells. J Immunol. (2017) 198:2172–81. 10.4049/jimmunol.1600165 28100682

[34] Mariño E Richards JL McLeod KH Stanley D Yap YA Knight J Gut microbial metabolites limit the frequency of autoimmune T cells and protect against type 1 diabetes. Nat Immunol. (2017) 18:552–562. 10.1038/ni.3713 28346408

[35] Haase S Haghikia A Wilck N Müller DN Linker RA. Impacts of microbiome metabolites on immune regulation and autoimmunity. Immunology (2018) 154:230–8. 10.1111/imm.12933 29637999 PMC5980218

[36] Kalina U Koyama N Hosoda T Nuernberger H Sato K Hoelzer D. Enhanced production of IL-18 in butyrate-treated intestinal epithelium by stimulation of the proximal promoter region. Eur J Immunol. (2002) 32:2635–43. 10.1002/1521-4141(200209)32:9<2635::AID-IMMU2635>3.0.CO;2-N 12207348

[37] Schauber J Svanholm C Termén S Iffland K Menzel T Scheppach W. Expression of the cathelicidin LL-37 is modulated by short chain fatty acids in colonocytes: relevance of signalling pathways. Gut (2003) 52:735–41. 10.1136/gut.52.5.735 12692061 PMC1773650

[38] Schauber J Iffland K Frisch S Kudlich T Schmausser B Eck M. Histone-deacetylase inhibitors induce the cathelicidin LL-37 in gastrointestinal cells. Mol Immunol. (2004) 41:847–54. 10.1016/j.molimm.2004.05.005 15261456

[39] Klaasen HL Van der Heijden PJ Stok W Poelma FG Koopman JP Van den Brink ME. Apathogenic, intestinal, segmented, filamentous bacteria stimulate the mucosal immune system of mice. Infect Immun. (1993) 61:303–6. 8418051 10.1128/iai.61.1.303-306.1993 PMC302719

[40] Talham GL Jiang HQ Bos NA Cebra JJ. Segmented filamentous bacteria are potent stimuli of a physiologically normal state of the murine gut mucosal immune system. Infect Immun. (1999) 67:1992–2000. 10085047 10.1128/iai.67.4.1992-2000.1999 PMC96557

[41] Ivanov II Atarashi K Manel N Brodie EL Shima T Karaoz U. Induction of intestinal Th17 cells by segmented filamentous bacteria. Cell (2009) 139:485–98. 10.1016/j.cell.2009.09.033 19836068 PMC2796826

[42] Gaboriau-Routhiau V Rakotobe S Lécuyer E Mulder I Lan A Bridonneau C. The key role of segmented filamentous bacteria in the coordinated maturation of gut helper T cell responses. Immunity (2009) 31:677–89. 10.1016/j.immuni.2009.08.020 19833089

[43] Wang J Li F Wei H Lian Z-X Sun R Tian Z. Respiratory influenza virus infection induces intestinal immune injury via microbiota-mediated Th17 cell-dependent inflammation. J Exp Med. (2014) 211:2397–410. 10.1084/jem.20140625 25366965 PMC4235643

[44] Deriu E Boxx GM He X Pan C Benavidez SD Cen L. Influenza Virus Affects Intestinal Microbiota and Secondary Salmonella Infection in the Gut through Type I Interferons. PLoS Pathog. (2016) 12:e1005572. 10.1371/journal.ppat.1005572 27149619 PMC4858270

[45] Bartley JM Zhou X Kuchel GA Weinstock GM Haynes L. Impact of age, caloric restriction, and influenza infection on mouse gut microbiome: an exploratory study of the role of age-related microbiome changes on influenza responses. Front Immunol. (2017) 8:1164. 10.3389/fimmu.2017.01164 28979265 PMC5611400

[46] Yildiz S Mazel-Sanchez B Kandasamy M Manicassamy B Schmolke M. Influenza A virus infection impacts systemic microbiota dynamics and causes quantitative enteric dysbiosis. Microbiome (2018) 6:9. 10.1186/s40168-017-0386-z 29321057 PMC5763955

[47] Groves HT Cuthbertson L James P Moffatt MF Cox MJ Tregoning JS. Respiratory disease following viral lung infection alters the murine gut microbiota. Front Immunol. (2018) 9:182. 10.3389/fimmu.2018.00182 29483910 PMC5816042

[48] Wu J Meng Z Jiang M Zhang E Trippler M Broering R. Toll-like receptor-induced innate immune responses in non-parenchymal liver cells are cell type-specific. Immunology (2010) 129:363–74. 10.1111/j.1365-2567.2009.03179.x 19922426 PMC2826681

[49] Ley RE Turnbaugh PJ Klein S Gordon JI. Microbial ecology: human gut microbes associated with obesity. Nature (2006) 444:1022–3. 10.1038/4441022a 17183309

[50] Ruiz A Cerdó T Jáuregui R Pieper DH Marcos A Clemente A One-year calorie restriction impacts gut microbial composition but not its metabolic performance in obese adolescents. Environ Microbiol. (2017) 19:1536–51. 10.1111/1462-2920.13713 28251782

[51] Ichinohe T Pang IK Kumamoto Y Peaper DR Ho JH Murray TS. Microbiota regulates immune defense against respiratory tract influenza A virus infection. Proc Natl Acad Sci USA. (2011) 108:5354–9. 10.1073/pnas.1019378108 21402903 PMC3069176

[52] Abt MC Osborne LC Monticelli LA Doering TA Alenghat T Sonnenberg GF. Commensal bacteria calibrate the activation threshold of innate antiviral immunity. Immunity (2012) 37:158–70. 10.1016/j.immuni.2012.04.011 22705104 PMC3679670

[53] Oh JZ Ravindran R Chassaing B Carvalho FA Maddur MS Bower M. TLR5-mediated sensing of gut microbiota is necessary for antibody responses to seasonal influenza vaccination. Immunity (2014) 41:478–92. 10.1016/j.immuni.2014.08.009 25220212 PMC4169736

[54] Gauguet S D'Ortona S Ahnger-Pier K Duan B Surana NK Lu R. Intestinal Microbiota of Mice Influences Resistance to Staphylococcus aureus Pneumonia. Infect Immun. (2015) 83:4003–14. 10.1128/IAI.00037-15 26216419 PMC4567647

[55] Schuijt TJ Lankelma JM Scicluna BP de Sousa e Melo F Roelofs JJTH de Boer JD. The gut microbiota plays a protective role in the host defence against pneumococcal pneumonia. Gut (2016) 65:575–83. 10.1136/gutjnl-2015-309728 26511795 PMC4819612

[56] Clarke TB Davis KM Lysenko ES Zhou AY Yu Y Weiser JN. Recognition of peptidoglycan from the microbiota by Nod1 enhances systemic innate immunity. Nat Med. (2010) 16:228–31. 10.1038/nm.2087 20081863 PMC4497535

[57] de Vrese M Winkler P Rautenberg P Harder T Noah C Laue C Probiotic bacteria reduced duration and severity but not the incidence of common cold episodes in a double blind, randomized, controlled trial. Vaccine (2006) 24:6670–4. 10.1016/j.vaccine.2006.05.048 16844267

[58] Vouloumanou EK Makris GC Karageorgopoulos DE Falagas ME. Probiotics for the prevention of respiratory tract infections: a systematic review. Int J Antimicrob Agents (2009) 34:197.e1–10. 10.1016/j.ijantimicag.2008.11.005 19179052

[59] Steed AL Christophi GP Kaiko GE Sun L Goodwin VM Jain U. The microbial metabolite desaminotyrosine protects from influenza through type I interferon. Science (2017) 357:498–502. 10.1126/science.aam5336 28774928 PMC5753406

[60] Maeda N Nakamura R Hirose Y Murosaki S Yamamoto Y Kase T. Oral administration of heat-killed Lactobacillus plantarum L-137 enhances protection against influenza virus infection by stimulation of type I interferon production in mice. Int Immunopharmacol. (2009) 9:1122–5. 10.1016/j.intimp.2009.04.015 19410659

[61] Hori T Kiyoshima J Shida K Yasui H. Augmentation of cellular immunity and reduction of influenza virus titer in aged mice fed Lactobacillus casei strain Shirota. Clin Diagn Lab Immunol. (2002) 9:105–8. 10.1128/CDLI.9.1.105-108.2002 11777838 PMC119906

[62] Wu S Jiang Z-Y Sun Y-F Yu B Chen J Dai C-Q. Microbiota regulates the TLR7 signaling pathway against respiratory tract influenza A virus infection. Curr Microbiol. (2013) 67:414–22. 10.1007/s00284-013-0380-z 23677145

[63] Trompette A Gollwitzer ES Pattaroni C Lopez-Mejia IC Riva E Pernot J. Dietary fiber confers protection against flu by shaping Ly6c- patrolling monocyte hematopoiesis and CD8+ T cell metabolism. Immunity (2018) 48:992–1005.e8. 10.1016/j.immuni.2018.04.022 29768180

[64] Sun K Metzger DW. Inhibition of pulmonary antibacterial defense by interferon-gamma during recovery from influenza infection. Nat Med. (2008) 14:558–64. 10.1038/nm1765 18438414

[65] Shahangian A Chow EK Tian X Kang JR Ghaffari A Liu SY. Type I IFNs mediate development of postinfluenza bacterial pneumonia in mice. J Clin Invest. (2009) 119:1910–20. 10.1172/JCI35412 19487810 PMC2701856

[66] Brown RL Sequeira RP Clarke TB. The microbiota protects against respiratory infection via GM-CSF signaling. Nat Commun. (2017) 8:1512. 10.1038/s41467-017-01803-x 29142211 PMC5688119

[67] Frank DN Feazel LM Bessesen MT Price CS Janoff EN Pace NR. The human nasal microbiota and Staphylococcus aureus carriage. PLoS ONE (2010) 5:e10598. 10.1371/journal.pone.0010598 20498722 PMC2871794

[68] Lemon KP Klepac-Ceraj V Schiffer HK Brodie EL Lynch SV Kolter R. Comparative analyses of the bacterial microbiota of the human nostril and oropharynx. MBio (2010) 1:e00129–10. 10.1128/mBio.00129-10 20802827 PMC2925076

[69] Bassis CM Tang AL Young VB Pynnonen MA. The nasal cavity microbiota of healthy adults. Microbiome (2014) 2:27. 10.1186/2049-2618-2-27 25143824 PMC4138944

[70] Charlson ES Bittinger K Haas AR Fitzgerald AS Frank I Yadav A. Topographical continuity of bacterial populations in the healthy human respiratory tract. Am J Respir Crit Care Med. (2011) 184:957–63. 10.1164/rccm.201104-0655OC 21680950 PMC3208663

[71] Yi H Yong D Lee K Cho Y-J Chun J. Profiling bacterial community in upper respiratory tracts. BMC Infect Dis. (2014) 14:583. 10.1186/s12879-014-0583-3 25391813 PMC4236460

[72] Ling Z Liu X Luo Y Yuan L Nelson KE Wang Y. Pyrosequencing analysis of the human microbiota of healthy Chinese undergraduates. BMC Genomics (2013) 14:390. 10.1186/1471-2164-14-390 23758874 PMC3685588

[73] Allen EK Koeppel AF Hendley JO Turner SD Winther B Sale MM. Characterization of the nasopharyngeal microbiota in health and during rhinovirus challenge. Microbiome (2014) 2:22. 10.1186/2049-2618-2-22 25028608 PMC4098959

[74] Dickson RP Erb-Downward JR Freeman CM McCloskey L Falkowski NR Huffnagle GB. Bacterial topography of the healthy human lower respiratory tract. MBio (2017) 8:e02287–16. 10.1128/mBio.02287-16 28196961 PMC5312084

[75] Abreu NA Nagalingam NA Song Y Roediger FC Pletcher SD Goldberg AN. Sinus microbiome diversity depletion and Corynebacterium tuberculostearicum enrichment mediates rhinosinusitis. Sci Transl Med. (2012) 4:151ra124. 10.1126/scitranslmed.3003783 22972842 PMC4786373

[76] Bassis CM Erb-Downward JR Dickson RP Freeman CM Schmidt TM Young VB. Analysis of the upper respiratory tract microbiotas as the source of the lung and gastric microbiotas in healthy individuals. MBio (2015) 6:e00037. 10.1128/mBio.00037-15 25736890 PMC4358017

[77] Venkataraman A Bassis CM Beck JM Young VB Curtis JL Huffnagle GB. Application of a neutral community model to assess structuring of the human lung microbiome. MBio (2015) 6:e02284–14. 10.1128/mBio.02284-14 25604788 PMC4324308

[78] Morris A Beck JM Schloss PD Campbell TB Crothers K Curtis JL. Comparison of the respiratory microbiome in healthy nonsmokers and smokers. Am J Respir Crit Care Med. (2013) 187:1067–75. 10.1164/rccm.201210-1913OC 23491408 PMC3734620

[79] Segal LN Alekseyenko AV Clemente JC Kulkarni R Wu B Gao Z. Enrichment of lung microbiome with supraglottic taxa is associated with increased pulmonary inflammation. Microbiome (2013) 1:19. 10.1186/2049-2618-1-19 24450871 PMC3971609

[80] Dickson RP Erb-Downward JR Freeman CM McCloskey L Beck JM Huffnagle GB Curtis JL. Spatial variation in the healthy human lung microbiome and the adapted island model of lung biogeography. Ann Am Thorac Soc. (2015) 12:821–30. 10.1513/AnnalsATS.201501-029OC 25803243 PMC4590020

[81] Rogers GB Shaw D Marsh RL Carroll MP Serisier DJ Bruce KD. Respiratory microbiota: addressing clinical questions, informing clinical practice. Thorax (2015) 70:74–81. 10.1136/thoraxjnl-2014-205826 25035125 PMC4283665

[82] Rogers GB Hoffman LR Carroll MP Bruce KD. Interpreting infective microbiota: the importance of an ecological perspective. Trends Microbiol. (2013) 21:271–6. 10.1016/j.tim.2013.03.004 23598051 PMC3880558

[83] Wertheim HFL Melles DC Vos MC van Leeuwen W van Belkum A Verbrugh HA. The role of nasal carriage in Staphylococcus aureus infections. Lancet Infect Dis. (2005) 5:751–62. 10.1016/S1473-3099(05)70295-4 16310147

[84] Bogaert D De Groot R Hermans PWM. Streptococcus pneumoniae colonisation: the key to pneumococcal disease. Lancet Infect Dis. (2004) 4:144–54. 10.1016/S1473-3099(04)00938-7 14998500

[85] Wolter N Tempia S Cohen C Madhi SA Venter M Moyes J. High nasopharyngeal pneumococcal density, increased by viral coinfection, is associated with invasive pneumococcal pneumonia. J Infect Dis. (2014) 210:1649–57. 10.1093/infdis/jiu326 24907383

[86] Ishizuka S Yamaya M Suzuki T Takahashi H Ida S Sasaki T. Effects of rhinovirus infection on the adherence of Streptococcus pneumoniae to cultured human airway epithelial cells. J Infect Dis. (2003) 188:1928–39. 10.1086/379833 14673774

[87] Avadhanula V Rodriguez CA Devincenzo JP Wang Y Webby RJ Ulett GC. Respiratory viruses augment the adhesion of bacterial pathogens to respiratory epithelium in a viral species- and cell type-dependent manner. J Virol. (2006) 80:1629–36. 10.1128/JVI.80.4.1629-1636.2006 16439519 PMC1367158

[88] Wang JH Kwon HJ Jang YJ. Rhinovirus enhances various bacterial adhesions to nasal epithelial cells simultaneously. Laryngoscope (2009) 119:1406–11. 10.1002/lary.20498 19434681

[89] Safaeyan F Nahaei MR Seifi SJ Kafil HS Sadeghi J. Quantitative detection of Staphylococcus aureus, Streptococcus pneumoniae and Haemophilus influenzae in patients with new influenza A (H1N1)/2009 and influenza A/2010 virus infection. GMS Hyg Infect Control (2015) 10:Doc06. 10.3205/dgkh000249 25914868 PMC4399408

[90] Hirano T Kurono Y Ichimiya I Suzuki M Mogi G. Effects of influenza A virus on lectin-binding patterns in murine nasopharyngeal mucosa and on bacterial colonization. Otolaryngol Head Neck Surg. (1999) 121:616–21. 10.1016/S0194-5998(99)70068-9 10547482

[91] McCullers JA McAuley JL Browall S Iverson AR Boyd KL Henriques Normark B. Influenza enhances susceptibility to natural acquisition of and disease due to Streptococcus pneumoniae in ferrets. J Infect Dis. (2010) 202:1287–95. 10.1086/656333 20822454 PMC3249639

[92] Wadowsky RM Mietzner SM Skoner DP Doyle WJ Fireman P. Effect of experimental influenza A virus infection on isolation of Streptococcus pneumoniae and other aerobic bacteria from the oropharynges of allergic and nonallergic adult subjects. Infect Immun. (1995) 63:1153–7. 7890365 10.1128/iai.63.4.1153-1157.1995 PMC173127

[93] Wren JT Blevins LK Pang B King LB Perez AC Murrah KA. Influenza A virus alters pneumococcal nasal colonization and middle ear infection independently of phase variation. Infect Immun. (2014) 82:4802–12. 10.1128/IAI.01856-14 25156728 PMC4249341

[94] Nakamura S Davis KM Weiser JN. Synergistic stimulation of type I interferons during influenza virus coinfection promotes Streptococcus pneumoniae colonization in mice. J Clin Invest. (2011) 121:3657–65. 10.1172/JCI57762 21841308 PMC3163966

[95] Diavatopoulos DA Short KR Price JT Wilksch JJ Brown LE Briles DE. Influenza A virus facilitates Streptococcus pneumoniae transmission and disease. FASEB J. (2010) 24:1789–98. 10.1096/fj.09-146779 20097876

[96] Vu HTT Yoshida LM Suzuki M Nguyen HAT Nguyen CDL Nguyen ATT. Association between nasopharyngeal load of Streptococcus pneumoniae, viral coinfection, and radiologically confirmed pneumonia in Vietnamese children. Pediatr Infect Dis J. (2011) 30:11–8. 10.1097/INF.0b013e3181f111a2 20686433

[97] Takase H Nitanai H Yamamura E Otani T. Facilitated expansion of pneumococcal colonization from the nose to the lower respiratory tract in mice preinfected with influenza virus. Microbiol Immunol. (1999) 43:905–7. 10553684 10.1111/j.1348-0421.1999.tb01226.x

[98] Tong HH Fisher LM Kosunick GM DeMaria TF. Effect of adenovirus type 1 and influenza A virus on Streptococcus pneumoniae nasopharyngeal colonization and otitis media in the chinchilla. Ann Otol Rhinol Laryngol. (2000) 109:1021–7. 10.1177/000348940010901106 11089992

[99] Peltola VT Boyd KL McAuley JL Rehg JE McCullers JA. Bacterial sinusitis and otitis media following influenza virus infection in ferrets. Infect Immun. (2006) 74:2562–7. 10.1128/IAI.74.5.2562-2567.2006 16622191 PMC1459735

[100] Edouard S Million M Bachar D Dubourg G Michelle C Ninove L. The nasopharyngeal microbiota in patients with viral respiratory tract infections is enriched in bacterial pathogens. Eur J Clin Microbiol Infect Dis. (2018) 37:172533. 10.1007/s10096-018-3305-8 30033505

[101] Verkaik NJ Nguyen DT de Vogel CP Moll HA Verbrugh HA Jaddoe VWV. Streptococcus pneumoniae exposure is associated with human metapneumovirus seroconversion and increased susceptibility to *in vitro* HMPV infection. Clin Microbiol Infect. (2011) 17:1840–4. 10.1111/j.1469-0691.2011.03480.x 21883660

[102] DE Lastours V Malosh R Ramadugu K Srinivasan U Dawid S Ohmit S Co-colonization by Streptococcus pneumoniae and Staphylococcus aureus in the throat during acute respiratory illnesses. Epidemiol Infect. (2016) 144:1–13. 10.1017/S0950268816001473 27535335 PMC9150196

[103] Ni K Li S Xia Q Zang N Deng Y Xie X. Pharyngeal microflora disruption by antibiotics promotes airway hyperresponsiveness after respiratory syncytial virus infection. PLoS ONE (2012) 7:e41104. 10.1371/journal.pone.0041104 22844430 PMC3406033

[104] Wang J Li F Sun R Gao X Wei H Li L-J. Bacterial colonization dampens influenza-mediated acute lung injury via induction of M2 alveolar macrophages. Nat Commun. (2013) 4:2106. 10.1038/ncomms3106 23820884 PMC3715851

[105] Langevin S Pichon M Smith E Morrison J Bent Z Green R. Early nasopharyngeal microbial signature associated with severe influenza in children: a retrospective pilot study. J Gen Virol. (2017) 98:2425–37. 10.1099/jgv.0.000920 28884664 PMC7011740

[106] Lu H-F Li A Zhang T Ren Z-G He K-X Zhang H Disordered oropharyngeal microbial communities in H7N9 patients with or without secondary bacterial lung infection. Emerg Microbes Infect. (2017) 6:e112 10.1038/emi.2017.101 29259328 PMC5750457

[107] Tarabichi Y Li K Hu S Nguyen C Wang X Elashoff D. The administration of intranasal live attenuated influenza vaccine induces changes in the nasal microbiota and nasal epithelium gene expression profiles. Microbiome (2015) 3:74. 10.1186/s40168-015-0133-2 26667497 PMC4678663

[108] Leung RK-K Zhou J-W Guan W Li S-K Yang Z-F Tsui SK-W. Modulation of potential respiratory pathogens by pH1N1 viral infection. Clin Microbiol Infect. (2013) 19:930–5. 10.1111/1469-0691.12054 23167452

[109] Chaban B Albert A Links MG Gardy J Tang P Hill JE. Characterization of the upper respiratory tract microbiomes of patients with pandemic H1N1 influenza. PLoS ONE (2013) 8:e69559. 10.1371/journal.pone.0069559 23844261 PMC3699515

[110] Greninger AL Chen EC Sittler T Scheinerman A Roubinian N Yu G. A metagenomic analysis of pandemic influenza A (2009 H1N1) infection in patients from North America. PLoS ONE (2010) 5:e13381. 10.1371/journal.pone.0013381 20976137 PMC2956640

[111] Jacoby P Watson K Bowman J Taylor A Riley TV Smith DW Kalgoorlie Otitis Media Research Project Team. Modelling the co-occurrence of Streptococcus pneumoniae with other bacterial and viral pathogens in the upper respiratory tract. Vaccine (2007) 25:2458–64. 10.1016/j.vaccine.2006.09.020 17030494 PMC7173051

[112] van den Bergh MR Biesbroek G Rossen JWA de Steenhuijsen Piters WAA Bosch AATM van Gils EJM Associations between pathogens in the upper respiratory tract of young children: interplay between viruses and bacteria. PLoS ONE (2012) 7:e47711 10.1371/journal.pone.0047711 23082199 PMC3474735

[113] Rosas-Salazar C Shilts MH Tovchigrechko A Schobel S Chappell JD Larkin EK. Differences in the nasopharyngeal microbiome during acute respiratory tract infection with human rhinovirus and respiratory syncytial virus in infancy. J Infect Dis. (2016) 214:1924–8. 10.1093/infdis/jiw456 27923952 PMC5142087

[114] Bogaert D Keijser B Huse S Rossen J Veenhoven R van Gils E. Variability and diversity of nasopharyngeal microbiota in children: a metagenomic analysis. PLoS ONE (2011) 6:e17035. 10.1371/journal.pone.0017035 21386965 PMC3046172

[115] Pérez-Losada M Alamri L Crandall KA Freishtat RJ. Nasopharyngeal microbiome diversity changes over time in children with asthma. PLoS ONE (2017) 12:e0170543. 10.1371/journal.pone.0170543 28107528 PMC5249091

[116] Hassoun A Huff MD Weisman D Chahal K Asis E Stalons D. Seasonal variation of respiratory pathogen colonization in asymptomatic health care professionals: A single-center, cross-sectional, 2-season observational study. Am J Infect Control (2015) 43:865–70. 10.1016/j.ajic.2015.04.195 26052103 PMC7115326

[117] DeMuri GP Gern JE Eickhoff JC Lynch SV Wald ER. Dynamics of bacterial colonization with streptococcus pneumoniae, haemophilus influenzae, and moraxella catarrhalis during symptomatic and asymptomatic viral upper respiratory tract infection. Clin Infect Dis. (2018) 66:1045–53. 10.1093/cid/cix941 29121208 PMC6019034

[118] Karppinen S Teräsjärvi J Auranen K Schuez-Havupalo L Siira L He Q. Acquisition and transmission of streptococcus pneumoniae are facilitated during rhinovirus infection in families with children. Am J Respir Crit Care Med. (2017) 196:1172–80. 10.1164/rccm.201702-0357OC 28489454

[119] Hofstra JJ Matamoros S van de Pol MA de Wever B Tanck MW Wendt-Knol H. Changes in microbiota during experimental human Rhinovirus infection. BMC Infect Dis. (2015) 15:336. 10.1186/s12879-015-1081-y 26271750 PMC4659412

[120] Thors V Christensen H Morales-Aza B Vipond I Muir P Finn A. The effects of live attenuated influenza vaccine on nasopharyngeal bacteria in healthy 2 to 4 year olds. a randomized controlled trial. Am J Respir Crit Care Med. (2016) 193:1401–9. 10.1164/rccm.201510-2000OC 26742001 PMC4910891

[121] Mina MJ McCullers JA Klugman KP. Live attenuated influenza vaccine enhances colonization of Streptococcus pneumoniae and Staphylococcus aureus in mice. MBio (2014) 5:e01040–13. 10.1128/mBio.01040-13 24549845 PMC3944816

[122] Salk HM Simon WL Lambert ND Kennedy RB Grill DE Kabat BFet al. Taxa of the nasal microbiome are associated with influenza-specific IgA response to live attenuated influenza vaccine. PLoS ONE (2016) 11:e0162803. 10.1371/journal.pone.0162803 27643883 PMC5028048

[123] Planet PJ Parker D Cohen TS Smith H Leon JD Ryan C. Lambda interferon restructures the nasal microbiome and increases susceptibility to staphylococcus aureus superinfection. MBio (2016) 7:e01939–01915. 10.1128/mBio.01939-15 26861017 PMC4752601

[124] Molyneaux PL Mallia P Cox MJ Footitt J Willis-Owen SAG Homola D. Outgrowth of the bacterial airway microbiome after rhinovirus exacerbation of chronic obstructive pulmonary disease. Am J Respir Crit Care Med. (2013) 188:1224–31. 10.1164/rccm.201302-0341OC 23992479 PMC3863728

[125] Vareille M Kieninger E Edwards MR Regamey N. The airway epithelium: soldier in the fight against respiratory viruses. Clin Microbiol Rev. (2011) 24:210–29. 10.1128/CMR.00014-10 21233513 PMC3021210

[126] Pittet LA Hall-Stoodley L Rutkowski MR Harmsen AG. Influenza virus infection decreases tracheal mucociliary velocity and clearance of Streptococcus pneumoniae. Am J Respir Cell Mol Biol. (2010) 42:450–60. 10.1165/rcmb.2007-0417OC 19520922 PMC2848738

[127] Smith CM Kulkarni H Radhakrishnan P Rutman A Bankart MJ Williams G. Ciliary dyskinesia is an early feature of respiratory syncytial virus infection. Eur Respir J. (2014) 43:485–96. 10.1183/09031936.00205312 23520320

[128] Abramson JS Mills EL Giebink GS Quie PG. Depression of monocyte and polymorphonuclear leukocyte oxidative metabolism and bactericidal capacity by influenza A virus. Infect Immun. (1982) 35:350–5. 7054126 10.1128/iai.35.1.350-355.1982 PMC351036

[129] Didierlaurent A Goulding J Patel S Snelgrove R Low L Bebien M. Sustained desensitization to bacterial Toll-like receptor ligands after resolution of respiratory influenza infection. J Exp Med. (2008) 205:323–9. 10.1084/jem.20070891 18227219 PMC2271005

[130] McNamee LA Harmsen AG. Both influenza-induced neutrophil dysfunction and neutrophil-independent mechanisms contribute to increased susceptibility to a secondary Streptococcus pneumoniae infection. Infect Immun. (2006) 74:6707–21. 10.1128/IAI.00789-06 16982840 PMC1698099

[131] Astry CL Jakab GJ. Influenza virus-induced immune complexes suppress alveolar macrophage phagocytosis. J Virol. (1984) 50:287–292. 6708169 10.1128/jvi.50.2.287-292.1984 PMC255619

[132] Franke-Ullmann G Pförtner C Walter P Steinmüller C Lohmann-Matthes ML Kobzik L. Alteration of pulmonary macrophage function by respiratory syncytial virus infection *in vitro*. J Immunol. (1995) 154:268–80. 7995946

[133] Ghoneim HE Thomas PG McCullers JA. Depletion of alveolar macrophages during influenza infection facilitates bacterial superinfections. J Immunol. (2013) 191:1250–9. 10.4049/jimmunol.1300014 23804714 PMC4907362

[134] Jakab GJ. Immune impairment of alveolar macrophage phagocytosis during influenza virus pneumonia. Am Rev Respir Dis. (1982) 126:778–82. 10.1164/arrd.1982.126.5.778 7149441

[135] Kleinerman ES Daniels CA Polisson RP Snyderman R. Effect of virus infection on the inflammatory response. Depression of macrophage accumulation in influenza-infected mice. Am J Pathol. (1976) 85:373–82. 11695 PMC2032573

[136] Nickerson CL Jakab GJ. Pulmonary antibacterial defenses during mild and severe influenza virus infection. Infect Immun. (1990) 58:2809–14. 2143751 10.1128/iai.58.9.2809-2814.1990 PMC313571

[137] Uehara Y Nakama H Agematsu K Uchida M Kawakami Y Abdul Fattah AS. Bacterial interference among nasal inhabitants: eradication of Staphylococcus aureus from nasal cavities by artificial implantation of Corynebacterium sp. J Hosp Infect. (2000) 44:127–33. 10.1053/jhin.1999.0680 10662563

[138] Iwase T Uehara Y Shinji H Tajima A Seo H Takada K. Staphylococcus epidermidis Esp inhibits Staphylococcus aureus biofilm formation and nasal colonization. Nature (2010) 465:346–9. 10.1038/nature09074 20485435

[139] Yan M Pamp SJ Fukuyama J Hwang PH Cho D-Y Holmes S. Nasal microenvironments and interspecific interactions influence nasal microbiota complexity and S. aureus carriage. Cell Host Microbe (2013) 14:631–40. 10.1016/j.chom.2013.11.005 24331461 PMC3902146

[140] Kanmani P Clua P Vizoso-Pinto MG Rodriguez C Alvarez S Melnikov V. Respiratory commensal bacteria corynebacterium pseudodiphtheriticum improves resistance of infant mice to respiratory syncytial virus and streptococcus pneumoniae superinfection. Front Microbiol. (2017) 8:1613. 10.3389/fmicb.2017.01613 28878760 PMC5572367

[141] Reddinger RM Luke-Marshall NR Hakansson AP Campagnari AA. Host physiologic changes induced by influenza a virus lead to staphylococcus aureus biofilm dispersion and transition from asymptomatic colonization to invasive disease. MBio (2016) 7:CD007087. 10.1128/mBio.01235-16 27507829 PMC4981728

[142] Pettigrew MM Marks LR Kong Y Gent JF Roche-Hakansson H Hakansson AP. Dynamic changes in the Streptococcus pneumoniae transcriptome during transition from biofilm formation to invasive disease upon influenza A virus infection. Infect Immun. (2014) 82:4607–19. 10.1128/IAI.02225-14 25135685 PMC4249342

[143] Marks LR Davidson BA Knight PR Hakansson AP. Interkingdom signaling induces Streptococcus pneumoniae biofilm dispersion and transition from asymptomatic colonization to disease. MBio (2013) 4:e00438–13. 10.1128/mBio.00438-13 23882016 PMC3735180

[144] Reddinger RM Luke-Marshall NR Sauberan SL Hakansson AP Campagnari AA. Streptococcus pneumoniae modulates staphylococcus aureus biofilm dispersion and the transition from colonization to invasive disease. MBio (2018) 9:e02089–17. 10.1128/mBio.02089-17 29317512 PMC5760742

