# Supplementary material for: Respiratory Viral Infection-Induced Microbiome Alterations and Secondary Bacterial Pneumonia
Source: Front Immunol. 2018 Nov 16;9:2640. doi: 10.3389/fimmu.2018.02640 (PMC6250824; doi:10.3389/fimmu.2018.02640)
Supplement: Supplementary file 1 [file Table_1.pdf]

Table 1. Changes in the microbiome after viral infection

Location of microbiome or colonization	Type of evaluation	Without viral infections		virus infections or stimulation	Changes in the bacterial burden, diversity, and host immune response after viral infections	Reference
		Predominant phyla	Predominant genera			
nostril	qPCR	—	*S. pneumoniae* (54%)<br>*M. catarrharis* (33%)<br>*H. influenzae* (26%)	Mainly RV	↑*S. pneumoniae*<br>↑*M. catarrharis*<br>↑*H. influenzae*	DeMuri et al., 2017
nasopharynx	Bacterial culture	—	*M. catarrlaris* 30.0 - 51.7%<br>*S. pneumoniae* 27.7 - 50.3%<br>*S. aureus* 20.2 - 25.8%<br>*H. influenzae* 12.8 - 41.8%	RV<br>adenovirus	Positive association between:<br>-RV and *S. pneumoniae*, *H. influenzae*, and *M. catarrharis*<br>-adenovirus and *M. catarrharis*	Jacoby et al., 2007
nasopharynx	Bacterial culture	—	*M. catarrlaris* 66 - 80%<br>*S. pneumoniae* 50 - 70%<br>*H. influenzae* 34 - 60%<br>*S. aureus* 5 - 16%	20 respiratory viruses	Positive association between:<br>-*S. aureus* and influenza virus<br>-*S. pneumoniae* and RV and enterovirus<br>-*H. influenzae* and RV and RSV<br>-*M. catarrharis* and coronavirus and adenovirus	van den Bergh et al., 2012
nasopharynx	qPCR	—	*S. aureus* 5%<br>*S. pneumoniae* 11%<br>*H. influenzae* 10%	Influenza A	*S. aureus* 12%<br>*S. pneumoniae* 24%<br>*H. Influenzae* 32%	Safaeyan et al., 2015
nasopharynx	qPCR	—	*S. pneumoniae*<br>*M. catarrharis*<br>*H. influenzae*<br>*S. aureus*	LAIV	LAIV increased the nasal colonization density of *S. pneumoniae* in subjects who harbored this bacterium at the time of vaccination, and transiently increased rates of colonization by *H. influenza*	Thors et al., 2016
nasopharynx	16S rRNA sequence	—	—	RV<br>RSV	Significant differences in the nasopharyngeal microbiome between patients with RV and RSV	Rosas-Salazar et al., 2016
nasopharynx	16S rRNA sequence	—	—	Influenza A and B	↓*S. aureus*<br>↑*Prevotella*<br>↑*Streptobacillus*<br>↑*Porphyromonas*<br>↑*Granulicatella*<br>↑*Veillonella*<br>↑*Fusobacterium*<br>↑*Haemophilus*<br>-Increased bacterial diversity is associated with influenza severity	Langevin e al., 2017

Abbreviations: RV, rhinovirus; RSV, respiratory syncytial virus; LAIV, live attenuated influenza vaccine

Table 1 (continued)

Location of microbiome or colonization	Type of evaluation	Without viral infections		virus infections or stimulation	Changes in the bacterial burden, diversity, and host immune response after viral infections	Reference
		Predominant phyla	Predominant genera			
nasopharynx	16S rRNA sequence	Actinobacteria<br>Firmicutes<br>Proteobacteria	*Corynebacterium*<br>*Staphylococcus*<br>*Moraxella*	LAIV	↑*Staphylococcus*<br>↑*Bacteroides*<br>-Up-regulation of type I and II IFN-stimulated genes and activation of tpe I IFN-mediated antiviral response may promote the bacterial growth.	Tarabichi et al., 2015
nasopharynx	16S rRNA sequence	—	—	Influenza A pdm09	Actinobacteria<br>Firmicutes<br>Proteobacteria<br>-A trend towards increasing diversity with patient age	Chaban et al., 2013
*nasopharynx	Bacterial culture	—	—	LAIV	↑*S. pneumoniae*<br>↑*S. aureus*<br>Type I IFN was increased in the nasopharynx and bonchoalveolar lavage specimens.	Mina et al., 2014
nasopharynx	16S rRNA sequence	Firmicutes<br>Actinobacteria<br>Proteobacteria	*Alloiococcus*<br>*Corynebacterium*<br>*Staphylococcus*<br>*Haemophilus*<br>*Propionibacterium*<br>*Streptococcus*	RV	↓*Haemophilus*<br>↓*Neisseria*<br>↑*Propionibacterium*	Allen et al., 2014
nasopharynx	16S rRNA sequence	Proteobacteria<br>Firmicutes<br>Actinobacteria	*Streptococcus*<br>*Rothia*<br>*Prevotella*	Influenza A and B<br>RSV<br>RV<br>metapneumovirus	Decreased diversity indices with symptomatic respiratory infection.<br>↓Anaerobes<br>↓*Prevotella*<br>↑*S. aureus*<br>↑*H. Influenzae*<br>↑*S. Pneumoniae*<br>↑*M. Catarrharis*<br>↑*Dol osigranulum pigrum*<br>↑*Corynebacterium propinquum/pseudodiphtheriticum*	Edouard et al,. 2018

Abbreviations: RV, rhinovirus; RSV, respiratory syncytial virus; LAIV, live attenuated influenza vaccine; IFN, interferon; * Mouse study

Table 1 (continued)

Location of microbiome	Type of evaluation	Without viral infections		virus infections or stimulation	Changes in the bacterial burden, diversity, and host immune response after viral infections	Reference
		Predominant phyla	Predominant genera			
throat	16S rRNA sequence	Firmicutes<br>Bacteroidetes<br>Proteobacteria<br>Actinobacteria	*Streptococcus*<br>*Prevotella*<br>*Veillonella*<br>*Rothia*<br>*Haemophilus*	RV	↑*H. parainfluenzae*<br>↑*Neisseria subflava*<br>↑*S. aureus*	Hofstra et al., 2015
oropharynx	Bacterial culture<br>Automated mass spectrometry	Bacteroidetes<br>Proteobacteria<br>Firmicutes	*Prevotella*<br>*Acinetobacter*	Influenza A pdm09	↑*Pseudomonas*<br>↑*Acinetobacter*	Leung et al., 2013
oropharynx	16S rRNA sequence	Bacteroidetes<br>Proteobacteria<br>Firmicutes	*Haemophilus*<br>*Bacteroides*	Influenza A (H7N9)	Significantly increased diversity compared to healthy controls, particularly H7N9 patients with secondary bacterial pneumonia.	Lu et al., 2017
Mixed URT (nasopharynx, oropharynx, and sputum)	16S rRNA sequence	—	*S. pneumoniae*	Influenza<br>Parainfluenza<br>RV<br>RSV<br>Coronavirus<br>adenovirus<br>metapneumovirus	Decreased diversity indices during viral infection.<br>Following 6 types of oropharyngeal microbiome were detected;<br>*Streptococcus* + *Prevotella* + *Veillonella*<br>*Streptococcus* + *Haemophilus* + *Neisseria*<br>*Streptococcus*<br>*Moraxella*<br>*Haemophilus*<br>*Klebsiella*<br>-are not associated with virus type but are linked to the age of subjects.	Yi et al., 2014
LRT (induced sputum)	16S rRNA sequence	Firmicutes (65%)<br>Bacteroidetes (17%)<br>Proteobacteria (5%)	*Streptococcus* (42.5%)<br>*Veillonella* (15.2%)<br>*Prevotella* (15.0%)	RV	Six-fold increase in bacterial burden.<br>↑Proteobacteria (*H. influenzae*)<br>↑Neutrophil concentration<br>↑Neutrophil elastase level	Molyneaux et al., 2013
∗LRT	16S rRNA sequence	Firmicutes (90%)	*Lactobacillus*	Influenza A	Minor qualitative change in microbiota and no quantitative impact on bacterial colonization.<br>Robust antimicrobial host response and increased sensitivity to bacterial super-infection.	Yildiz et al., 2018

Abbreviations: RV, rhinovirus; RSV, respiratory syncytial virus ; IFN, interferon; LRT, lower respiratory tract; ∗ Mouse study

Table 1 (continued)

Location of microbiome	Type of evaluation	Without viral infections		virus infections or stimulation	Changes in the bacterial burden, diversity, and host immune response after viral infections	Reference
		Predominant phyla	Predominant genera			
∗Gut	16S rRNA sequence	Bacteroidetes (71%)	S24-7 (*Bacteroidia*)	Influenza A	Depletion of bacterial content (↓Bacteroidetes, ↑Firmicutes)<br>Disruption of mucus layer integrity and higher levels of antimicrobial peptides in Paneth cells.	Yildiz et al., 2018
∗Gut	16S rRNA sequence	Bacteroidetes<br>Firmicutes<br>Tenericutes<br>Actinobacteria<br>Proteobacteria<br>Deferribacteres	—	RSV<br>Influenza	↑Bacteroidetes<br>↓Firmicutes	Groves et al., 2018
∗Gut	16S rRNA sequence	Fimicutes<br>Bacteroidetes	—	Influenza A (PR8)	↑Proteobacteria	Bartley et al., 2017
∗Gut	16S rRNA sequence	Bacteroidetes<br>Firmicutes	S24-7 (*Bacteroidia*)	Influenza A (PR8)	↑Proteobacteria<br>-Influenza-induced antiviral type I IFN pathway is partly responsible for the inadequate innate immune cell responses during host defense against secondary bacterial infections.	Deriu et al., 2016
∗Gut	16S rRNA sequence	—	*Lactobacillus*<br>*Bacteroides*	Influenza A (PR8)	↓*Lactobacillus*<br>↑*Enterobacteriaceae*<br>-Influenza infection altered intestinal microbiota composition, which was mediated by type II IFN produced by lung-derived T cells recruited to the intestine.	Wang et al., 2014

Abbreviations: RSV, respiratory syncytial virus ; IFN, interferon; ∗ Mouse study
